# Genome-wide association of the metabolic shifts underpinning dark-induced senescence in Arabidopsis

**DOI:** 10.1093/plcell/koab251

**Published:** 2021-10-08

**Authors:** Feng Zhu, Saleh Alseekh, Kaan Koper, Hao Tong, Zoran Nikoloski, Thomas Naake, Haijun Liu, Jianbing Yan, Yariv Brotman, Weiwei Wen, Hiroshi Maeda, Yunjiang Cheng, Alisdair R Fernie

**Affiliations:** National R&D Center for Citrus Preservation, Key Laboratory of Horticultural Plant Biology, Ministry of Education, Huazhong Agricultural University, Wuhan 430070, China; Max-Planck-Institut für Molekulare Pflanzenphysiologie, Potsdam-Golm 14476, Germany; Max-Planck-Institut für Molekulare Pflanzenphysiologie, Potsdam-Golm 14476, Germany; Center of Plant Systems Biology and Biotechnology, Plovdiv 4000, Bulgaria; Department of Botany, University of Wisconsin–Madison, Madison, Wisconsin 53706, USA; Max-Planck-Institut für Molekulare Pflanzenphysiologie, Potsdam-Golm 14476, Germany; Center of Plant Systems Biology and Biotechnology, Plovdiv 4000, Bulgaria; Bioinformatics, Institute of Biochemistry and Biology, University of Potsdam, Potsdam 14476, Germany; Max-Planck-Institut für Molekulare Pflanzenphysiologie, Potsdam-Golm 14476, Germany; Center of Plant Systems Biology and Biotechnology, Plovdiv 4000, Bulgaria; Bioinformatics, Institute of Biochemistry and Biology, University of Potsdam, Potsdam 14476, Germany; Max-Planck-Institut für Molekulare Pflanzenphysiologie, Potsdam-Golm 14476, Germany; National Key Laboratory of Crop Genetic Improvement, Huazhong Agricultural University, Wuhan 430070, China; Gregor Mendel Institute, Austrian Academy of Sciences, Vienna 1030, Austria; National Key Laboratory of Crop Genetic Improvement, Huazhong Agricultural University, Wuhan 430070, China; Max-Planck-Institut für Molekulare Pflanzenphysiologie, Potsdam-Golm 14476, Germany; Department of Life Sciences, Ben-Gurion University of the Negev, Beersheba, Israel; National R&D Center for Citrus Preservation, Key Laboratory of Horticultural Plant Biology, Ministry of Education, Huazhong Agricultural University, Wuhan 430070, China; Department of Botany, University of Wisconsin–Madison, Madison, Wisconsin 53706, USA; National R&D Center for Citrus Preservation, Key Laboratory of Horticultural Plant Biology, Ministry of Education, Huazhong Agricultural University, Wuhan 430070, China; Max-Planck-Institut für Molekulare Pflanzenphysiologie, Potsdam-Golm 14476, Germany; Center of Plant Systems Biology and Biotechnology, Plovdiv 4000, Bulgaria

## Abstract

Dark-induced senescence provokes profound metabolic shifts to recycle nutrients and to guarantee plant survival. To date, research on these processes has largely focused on characterizing mutants deficient in individual pathways. Here, we adopted a time-resolved genome-wide association-based approach to characterize dark-induced senescence by evaluating the photochemical efficiency and content of primary and lipid metabolites at the beginning, or after 3 or 6 days in darkness. We discovered six patterns of metabolic shifts and identified 215 associations with 81 candidate genes being involved in this process. Among these associations, we validated the roles of four genes associated with glycine, galactinol, threonine, and ornithine levels. We also demonstrated the function of threonine and galactinol catabolism during dark-induced senescence. Intriguingly, we determined that the association between tyrosine contents and *TYROSINE AMINOTRANSFERASE 1* influences enzyme activity of the encoded protein and transcriptional activity of the gene under normal and dark conditions, respectively. Moreover, the single-nucleotide polymorphisms affecting the expression of *THREONINE ALDOLASE 1* and the amino acid transporter gene *AVT1B*, respectively, only underlie the variation in threonine and glycine levels in the dark. Taken together, these results allow us to present a very detailed model of the metabolic aspects of dark-induced senescence, as well as the process itself.

## Teaser

Time-resolved GWAS of *Fv/Fm*, chlorophyll, primary and lipid metabolites content allowed a comprehensive view of the genetic landscape of dark-induced senescence.

## Introduction

In cellular circumstances under which carbohydrates are scarce, plants can metabolize proteins and lipids as alternative respiratory substrates (Kunz et al., 2009; Araujo et al., 2011b). Dark-induced senescence is one such circumstance, being a highly coordinated process leading to the degradation of polymers and the subsequent recovery of nutrients for use in sink tissues. This process involves a clear shift from anabolic to catabolic processes in an attempt to sacrifice leaves for the sake of recycling carbon (C) and nitrogen (N) sources (Guo et al., 2004; Buchanan-Wollaston et al., 2005; Mueller-Roeber and Balazadeh, 2014; Kamranfar et al., 2018). In senescing leaves, this process begins with intracellular organellar degradation—first the chloroplasts and then the mitochondria and the nucleus (Gan, 2007; Taylor et al., 2010). This degradation order is not surprising, given that 70% of total leaf protein is housed within the chloroplast and that Rubisco and chlorophyll (Chl) *a*/*b* binding protein are the largest reservoirs of recoverable N in vegetative tissues (Makino and Osmond, 1991; Otegui et al., 2005). Autophagy, together with the newly uncovered chloroplast vesicularization pathway and senescence-associated vacuoles, play considerable roles in the degradation of chloroplast proteins during senescence (Otegui et al., 2005; Carrion et al., 2013; Wang and Blumwald, 2014; Barros et al., 2020). Destabilization of photosystems (PSs) alongside degradation of Chl *a*/*b* binding proteins release both free Chl and its toxic degradation products, the removal of which requires further degradation via the pheophorbide a oxygenase pathway, which has recently been comprehensively characterized (Hortensteiner, 2006, 2009; Hortensteiner and Krautler, 2011; Sakuraba et al., 2012).

Given the changes mentioned above, it is not surprising that photosynthesis is massively impaired and heterotrophic metabolism comes to force, leading to the following conundrum: senescing leaves rapidly become starved for C, yet, are highly energy-consuming with an active tricarboxylic acid (TCA) cycle being of critical importance. As such, proteins, lipids, and Chl provide alternative substrates for the TCA cycle, allowing the maintenance of high respiratory rates in the near absence of carbohydrates (Araujo et al., 2011a). In addition, catabolism of lysine, branched-chain and aromatic amino acids, and phytol by isovaleryl CoA dehydrogenase and 2-hydroxyglutarate dehydrogenase provide TCA cycle substrates but also directly channel electrons to the mitochondrial electron transport chain, with the degradation of amino acids being particularly vital in this respect (Ishizaki et al., 2005, 2006; Araujo et al., 2010; Engqvist et al., 2011). Recent findings have additionally pinpointed the involvement of transcription factors (such as PHYTOCHROME-INTERACTING FACTORs, RESPONSIVE TO DESICCATION 26, and ATAF2) in mediating the metabolic shifts occurring during senescence (Song et al., 2014; Kamranfar et al., 2018; Nagahage et al., 2020). Despite the fact that microarray data have allowed a broad view of the transcriptional responses to dark-induced senescence, an understanding of the large scale metabolic shifts of the nutrient recycling (such as amino acids, lipids, and sugars) to guarantee plant survival, which can give some cues to biofortify crop quality and stress resistance, remains fragmentary. Indeed, most studies to date have either looked at the regulation of protein or lipid degradation independently (Kunz et al., 2009; Araujo et al., 2011b; Luzarowska et al., 2020), while a comprehensive study to that of developmental-induced senescence has not yet been conducted (Watanabe and Lam, 2004). However, fairly complete surveys of the transcriptional and metabolic changes in autophagy-deficient mutants of Arabidopsis (*Arabidopsis thaliana*) have recently been reported (Avin-Wittenberg et al., 2015; Barros et al., 2017), with many shared metabolic features between these and the targeted studies described above. This observation suggests that there is, indeed, a crucial role for autophagy in dark-induced senescence.

Recently, metabolite-based genome-wide association study (mGWAS) has been demonstrated as a powerful method to identify genes involved in different pathways (Francisco et al., 2016; Zhu et al., 2018; Slaten et al., 2020). However, some metabolites pathways are only activated under specific stresses and their underlying genetic variation thus cannot be identified from GWAS data collected under normal conditions (nonstress). Moreover, given that the metabolite levels during stress change dynamically, GWAS based on several time points during stress may identify novel and dynamic changes of genetic variation for the observed traits under stress. To analyze the genomic landscape of the important parameters in dark-induced senescence, such as photochemical efficiency (maximum variable fluorescence/maximum yield of fluorescence [*Fv/Fm*]) and Chl content, and the levels of polar primary metabolites and lipids under darkness, we screened and carried out a time-resolved GWAS of a diversity panel of 252 Arabidopsis accessions before darkness and after extended darkness for 3and 6 days. While we observed no clear associations between either *Fv/Fm* or Chl content, we revealed several associations for lipid levels and many associations for amino acid levels. We further validated six of these associations using T-DNA insertion mutants and other means. Importantly, the mutants of four genes, *THREONINE ALDOLASE 1* (*THA1*), *TYROSINE AMINOTRANSFERASE 1* (*TAT1*), *BETA GLUCOSIDASE 42* (*BGLU42*), and *BRANCHED-CHAIN AMINO ACID TRANSAMINASE 2* (*BCAT2*), displayed compromised *Fv/Fm* and Chl abundance. We evaluate here their effects against dark-induced senescence and discuss these findings in the context of our current understanding of the regulation of metabolic pathways and with regard to models of dark-induced senescence. In addition, we more generally discuss the value of reverse genetic validation of candidate genes not only in confirming candidate genes but also in evaluating the consequences of more severe genetic interventions to elucidate the control of complex multigenic responses such as those underlying dark-induced senescence.

## Results

### Variation in photochemical efficiency and Chl content in Arabidopsis natural population during darkness

*Fv/Fm* and Chl content represent important parameters reflecting leaf senescence, prompting us to analyze these senescence-related traits first in samples collected at 0, 3, and 6 days into darkness. Following treatment in extended darkness, both *Fv/Fm* and Chl content substantially decreased, indicating darkness-induced rapid senescence (Supplemental Data Set S1). Moreover, Manhattan plots of these traits revealed that the highest associated single-nucleotide polymorphism (SNP) with *Fv/Fm* and Chl content only has a *P*-value = 0.644 × 10^−6^, with a significance threshold of ∼1 × 10^−6^. In agreement, no obvious candidate genes involved in either photochemical efficiency or Chl metabolism mapped within the genomic region with the lowest *P*-values (Supplemental Figure S1; Supplemental Data Sets S2–S4). Moreover, the broad-sense heritability (*H^2^*) of *Fv/Fm* and Chl content across the three time points was only 0.03 and 0.10 (Supplemental Data Set S5), respectively, indicating that the genetic effects on *Fv/Fm* and Chl content variation are quite low.

### Variation in primary and lipids metabolites among Arabidopsis accessions during darkness

To investigate the broad genetic and metabolic landscape of the Arabidopsis response to darkness stress, we determined the levels of primary metabolites and lipids across the diversity panel over two seasons at 0, 3, and 6 days into darkness. For this purpose, we identified 235 known metabolites by gas chromatography–mass spectrometry (GC–MS) and liquid chromatography–MS (LC–MS). While this approach will ensure that only genetically robust or canalized traits (Alseekh et al., 2017) can be mapped, it will clearly also lead to the exclusion of those that are influenced by the environment. To obtain more reliable data for the GWAS analysis, we set stringent criteria to select both metabolites and accessions: only those accessions harvested at all three time points and both seasons, and those metabolites identified among all selected accessions for at least one time point, were included in downstream data processing. Following these cutoffs, we used 136 detected metabolites from 252 Arabidopsis accessions for all analyses: 22 amino acids, 13 organic acids, 8 sugars, 3 amines, 3 diacylglycerols (DAGs), 12 digalactosyldiacylglycerols (DGDGs), 13 monogalactosyldiacylglycerols (MGDGs), 17 phosphatidylcholines (PCs), 10 phosphatidylethanolamines (PEs), 27 triacylglycerides (TAGs), 2 sulfoquinovosyldiacylglycerol, and 6 other metabolites (Supplemental Data Set S1).

**Figure 1:**
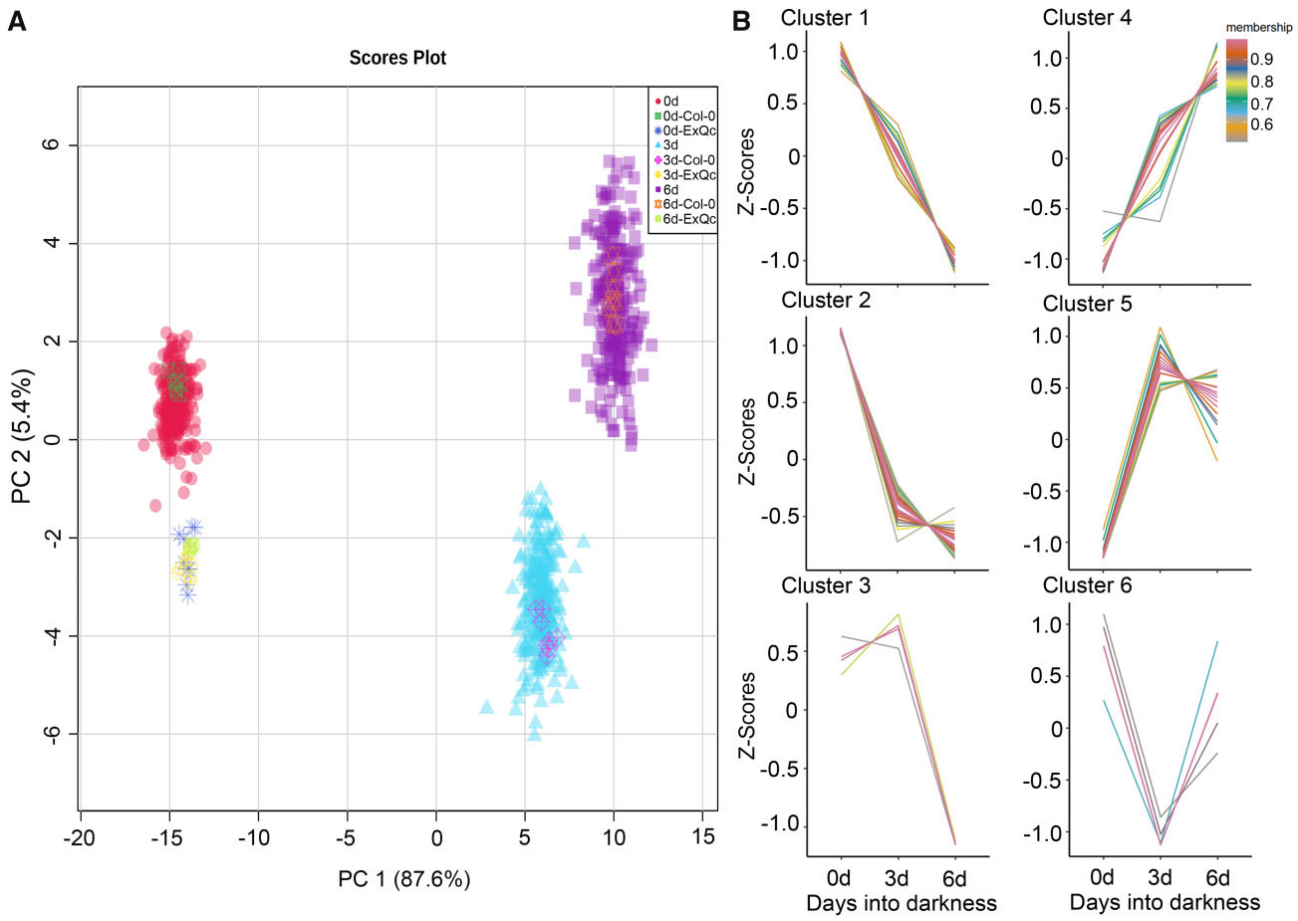
Variation in the Arabidopsis metabolome upon darkness treatment. A, Principle component analysis of metabolite levels of 252 Arabidopsis accessions for three time points. B, Different patterns of metabolite levels across the three time points.

A principal component analysis (PCA) of the different time points and accessions with all metabolites illustrated the separation of the 0 days samples from the 3 and 6 days samples, suggesting that darkness contributes more to the shifts in metabolite abundance than the genotypic differences (Figure 1A). This result was in agreement with the low broad-sense heritability of the metabolites across the three conditions. Just three metabolites (or 2.2% of total) displayed a heritability >0.60, while 8 metabolites (5.9% of total) had a heritability between 0.40 and 0.60, and the remaining 125 (91.9%) metabolites showed a heritability ˂0.40 among the three conditions (Supplemental Data Set S5). However, the control samples used to assess extraction quality control (Ex-QC) of different time points closely clustered, validating the high quality of the data normalization pipeline and the accuracy of the analysis concerning the different metabolite intensities between time points (Figure 1A).

We then used the average value of each metabolite across all accessions at the three time points to analyze their dynamics upon exposure to darkness, forming six dynamic patterns. Of those, Clusters 1, 2, 4, and 5 comprise 91.26% of all metabolites. Clusters 1 and 2 contained sugars and sugar alcohols (such as trehalose, galactinol, sucrose, fructose, and glucose), TCA cycle metabolites (such as fumaric acid, citric acid, and malic acid), amino acids, and galactolipids (MGDGs and DGDGs; Supplemental Data Set S6). Moreover, the levels of some metabolites (such as ornithine, glutamine, glutamate, and asparagine) which play important roles in N recycling and remobilization, continually increased during darkness (Cluster 4). Interestingly, branched-chain amino acids (BCAAs; isoleucine, leucine, and valine), tyrosine, lysine, threonine, and PCs, TAGs belonged to Cluster 5, with higher levels from 0 to 3 days, followed by a decrease at 6 days (Cluster 5) (Figure 1B; Supplemental Data Set S6), a pattern that reflected the degradation of macromolecules at the early stage of darkness stress and their later utilization as respiratory substrates during extended darkness.

### Genetic basis of the variation in the Arabidopsis metabolome in different datasets

**Figure 2:**
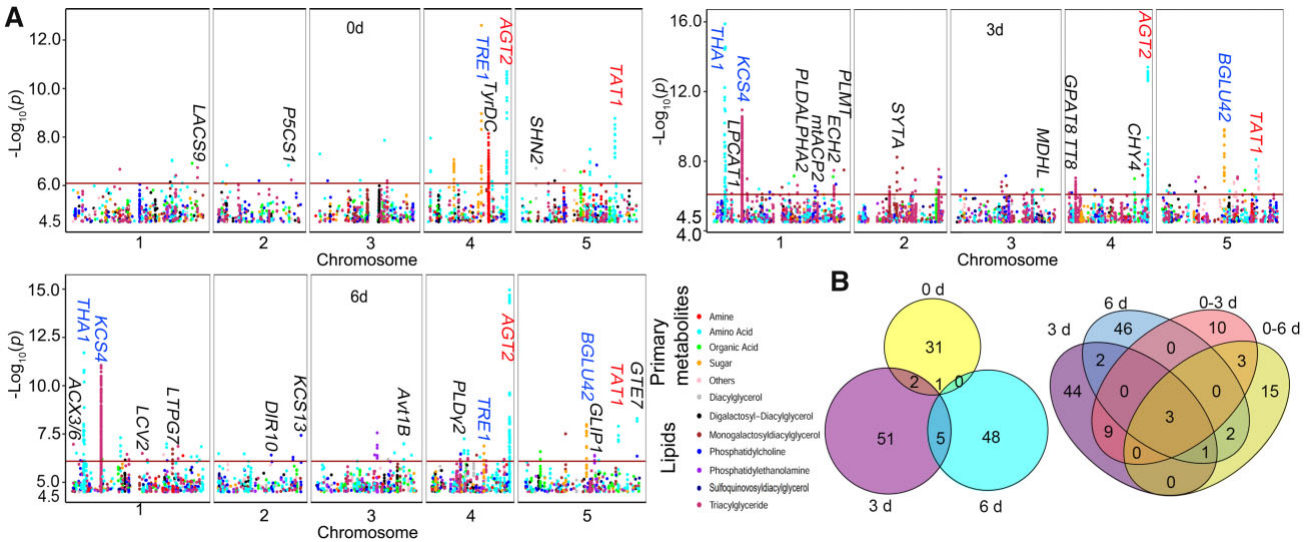
Overview of mGWAS results in different datasets. A, Manhattan plots of mGWAS results for the different datasets. Red horizontal lines in the Manhattan plots indicate the genome-wide significance threshold (*P*-value = 0.81 × 10^−6^). Only the genes that have been functionally characterized and are most closely linked to the significant GWAS associations mapped in this study are marked above the signal peaks (black, blue, and red colors indicate genes identified in one, two, or three datasets, respectively). B, Venn diagram showing the extent of overlap between detected SNPs by GWAS for the 0, 3, and 6 days datasets and the dark-related datasets (3, 6, 0–3, and 0–6 days) exceeding the genome-wide threshold.

We then performed GWAS by integrating all metabolite contents obtained with the imputed 1.2-M SNP information on the Arabidopsis accessions (Arouisse et al., 2020), using a mixed linear model for each individual time point (0, 3, and 6 days) as well as the differences in metabolite levels between the different time points (0–3, 3–6, and 0–6 days). The GWAS results indicated that 81 metabolites from the 6 datasets analyzed here are associated with at least one locus exceeding the genome-wide threshold (*P*-value = 8.10 × 10^−7^). In detail, we identified 34 (0 days), 59 (3 days), 54 (6 days), 25 (0–3 days), 19 (3–6 days), and 24 (0–6 days) loci exceeding the genome-wide threshold (Figure 2, A and B; Supplemental Data Set S2). Moreover, we determined that 81 genes in these associations may be directly involved in primary and lipid metabolism, based on the validation carried out in this study or existing functional annotation information (Supplemental Data Set S2; http://aralip.plantbiology.msu.edu/). Additionally, to compare differences across time points, we repeated GWAS based on the multivariate models; as the imputation of the SNPs used here excluded some SNPs (and thus their associations) from the 200 K SNPs represented by the Affymetrix Chip, we performed another GWAS based on the Affymetrix Chip SNP data using the mixed linear model. Notably, GWAS results obtained with the multivariate models and the Affymetrix Chip data were similar to the 1.2-M SNP results, especially in the case of strong associations. We, therefore, used the GWAS results based on individual time points. However, because the imputation excluded some interesting SNPs, we will also describe associations, such as that of *BCAT2* with leucine, *PROLINE DEHYDROGENASE 1* (*ProDH1*) with proline and *ASPARTATE OXIDASE* (*AO*)*/FORMATE DEHYDROGENASE* (*FDH*) with nicotinic acid, solely based on the 200 K SNPs from the Affymetrix Chip data (Supplemental Figure S2). The detailed results based on multivariate GWAS models and the 200 K Affymetrix SNPs are provided in Supplemental Data Sets S3 and S4, respectively.

The PCA suggested only small differences between the 3 and 6 days samples (Figure 1A). In agreement, the correlation-based metabolite networks based on the individual and different metabolite levels across the three time points revealed that the 3–6 days metabolite network differs from the 0–3 days and 0–6 days networks (Supplemental Figure S3). Moreover, GWAS results also indicated that the associated loci identified for the 3–6 days dataset are different from those associated with the other dark-related datasets (3, 6, 0–3, and 0–6 days) (Supplemental Data Set S2). Therefore, to identify darkness-associated loci, we only focused on those associated loci in the 3, 6, 0–3, and 0–6 days datasets and analyzed the corresponding overlapping loci, which may represent the most important loci responding to extended darkness. We thus identified three loci in all four datasets that are associated with tyrosine, threonine, and TAG 54:9 levels; we also identified one locus associated with β-alanine levels in the 3, 6, and 0–6 days datasets. In addition, one locus associated with galactinol levels (3 and 6 days datasets), one locus associated with trehalose levels (0–3 and 0–6 days datasets) and two loci associated with ornithine and glycine levels (0–6 and 6 days datasets) exhibited strong associations in two datasets (Figure 2B; Supplemental Data Set S7). Based on the clustering of metabolites over the time course, glycine, galactinol, and trehalose belonged to Clusters 1 and 2, with a decreasing abundance pattern; ornithine belonged to Cluster 4, with an increasing abundance, while tyrosine, threonine, β-alanine, and TAG 54:9 belonged to Cluster 5, with a peak in abundance 3 days into darkness (Figure 1B; Supplemental Data Set S6). Therefore, we further analyzed the genetic mechanism underpinning the levels of these metabolites to explore the regulation of Arabidopsis against starvation stress brought upon by extended darkness.

### Genetic regulation of galactinol, trehalose, and glycine levels

Considering that the shift in metabolite pattern may reflect their function, the decreasing pattern seen for the metabolites that included sugars, sugar alcohols, and organic acids was in agreement with their role as direct energy source under C starvation caused by darkness (Figure 1B; Supplemental Data Set S6). The GWAS analysis identified some interesting associations, such as between citric acid and SNP-Chr1:27902164 near At1g74240 (*MITOCHONDRIAL SUBSTRATE CARRIER FAMILY PROTEIN*) locus (*P*-value = 7.81 × 10^−8^) within the 3 days dataset (Figure 2A; Supplemental Data Set S2). Moreover, the levels of several amino acids and galactolipids (MGDGs and DGDGs) also exhibited a decreasing pattern. We observed associations between SNPs located close to attractive candidate genes that were in agreement with their function in the related metabolite pathways (Routaboul et al., 1999; Aharoni et al., 2004; Qin et al., 2006; Launay et al., 2019; Supplemental Figure S4). Specifically, SNP Chr2:16607915 around the At2g39800 (*DELTA1-PYRROLINE-5-CARBOXYLATE SYNTHASE 1* [*P5CS1*]) locus showed an association with proline levels (*P*-value = 1.46 × 10^−7^ and 7.15 × 10^−7^) in the 0 and 0–6 days datasets; SNP Chr5:3561864 around the At5g11190 (*SHINE2* [*SHN2*]) locus was associated with DAG 34:2 levels (*P*-value = 1.93 × 10^−7^) in the 0 days dataset; SNP Chr4:7102376 close to the At4g11830 (*PHOSPHOLIPASE D GAMMA 2* [*PLDγ2*]) locus was associated with PC 32:1 (2) levels (*P*-value = 3.36 × 10^−7^) in the 6 days dataset; finally, SNP Chr2:8437156 close to the At2g19450 (*TAG1*) locus was associated with DAG 36:5 levels (*P*-value = 7.11 × 10^−7^) in the 0–3 days dataset. Furthermore, the levels of tyramine, the product of tyrosine decarboxylation, decreased during darkness and was associated with SNP Chr4:14157881 near At4g28680 (*TYROSINE DECARBOXYLASE* [*TyrDC*]) (*P*-value = 7.23 × 10^−9^), which was also previously reported (Wu et al., 2016). Besides these associations, three loci (identified in at least two datasets) exhibited strong association with galactinol, trehalose, and glycine levels, which we analyzed further below.

**Figure 3:**
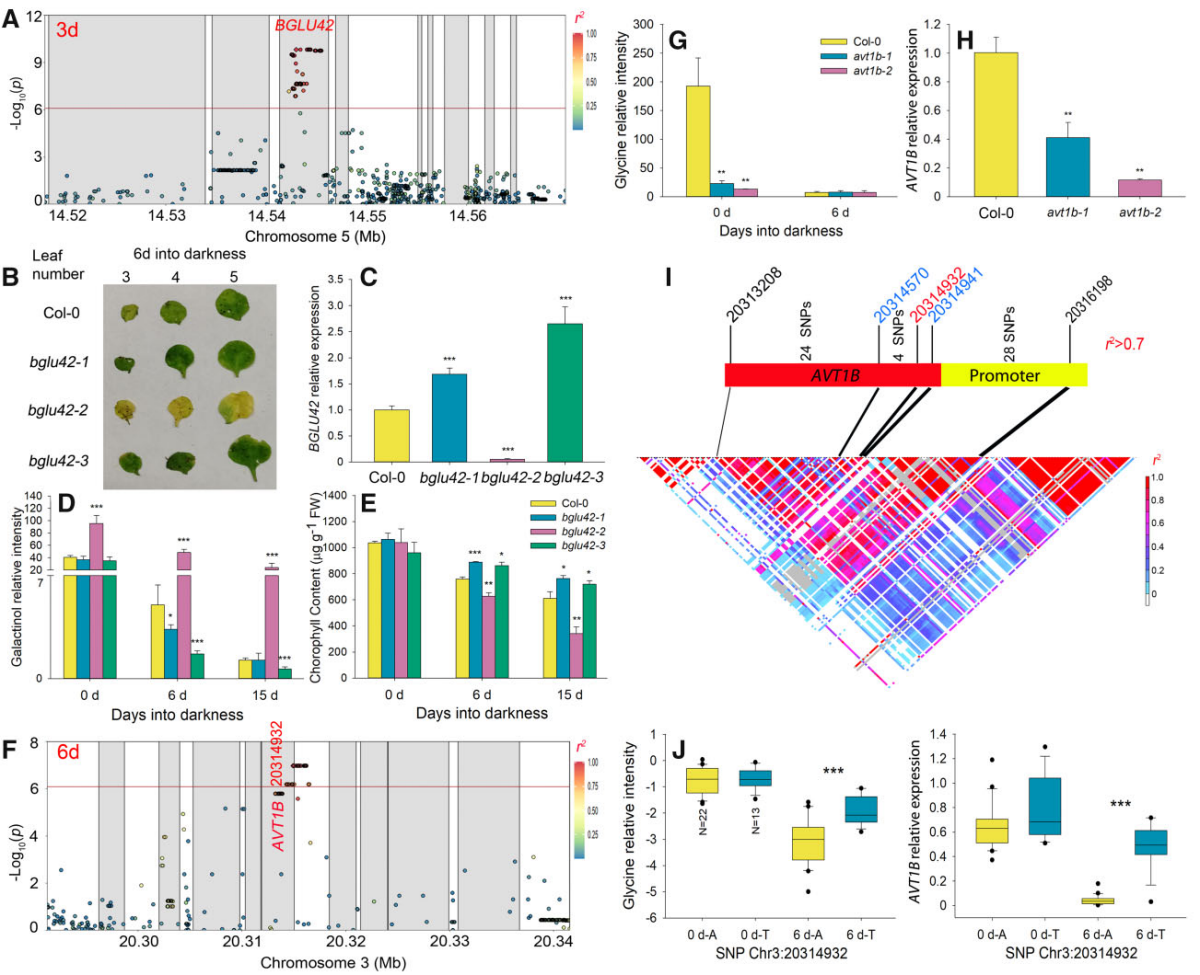
Variation at *BGLU42* and *AVT1B* affects galactinol and glycine contents under extended darkness. A, LD plot based on the imputed 1.2 M SNP data for the association with galactinol levels for the 3 days dataset. The *x*-axis shows the physical positions in Mb and the *y*-axis indicates the significance level of each SNP as Log_10_(*P*-value). Each gray block denotes a gene and each circle indicates an SNP, with the lead SNP (with lowest *P*-value) shown as a red diamond. The color of each circle reflects the *r^2^* value with the lead SNP. B–E, Phenotypes of *bglu42-1*, *bglu42-2* and *bglu42-3* mutants upon extended darkness. B, Relative *BGLU42* expression levels in Col-0, *bglu42-1*, *bglu42-2*, and *bglu42-3* mutant seedlings, as determined by RT-qPCR. Data are shown as means ± standard deviation (sd; *n* = 6 biological replicates). C, Dark-induced senescence phenotype. D, Galactinol content and (E) Chl content of Col-0, *bglu42-1*, *bglu42-2*, and *bglu42-3* mutants. Data are shown as means ± sd (*n* = 3 biological replicates). Asterisks indicate statistically significant differences relative to Col-0, as determined by two-tailed Student’s *t* test: *P < 0.05; **P < 0.01; ***P < 0.001. F, LD plot based on imputed 1.2 M SNP data for the association with glycine levels for the 6 days dataset. The *x*- and *y*-axes are as in (A). Each gray block denotes a gene and each circle indicates an SNP, with the lead SNP (with lowest *P*-value) shown as a red diamond. The color of each circle reflects the *r^2^* value with the lead SNP. G and H, Phenotype of *avt1b-1* and *avt1b-2* mutants. G, Relative *AVT1B* expression levels in Col-0, *avt1b-1*, and *avt1b-2* mutants, as determined by RT-qPCR. H, Glycine content of Col-0, *avt1b-1*, and *avt1b-2* mutants in normal conditions and upon extended darkness. For (G) and (H), data are shown as means ± sd (*n* = 6 biological replicates). Asterisks indicate statistically significant differences relative to Col-0, as determined by two-tailed Student’s *t* test: ***P < 0.001. I, LD plot based on the SNPs around the *AVT1B* gene region from the 1,001 Arabidopsis genomes database. The lead SNP is shown in red. The SNPs in the gene body that change AVT1B protein sequence are shown in blue. J, Glycine content (left) and relative *AVT1B* expression levels (right) of randomly selected accessions, shown as boxplots. The number of accessions with each genotype is given for the 0 days phenotypic values. The boxplots represent the interquartile range, the solid horizontal line represents the median, the whiskers represent 1.5× interquartile range, and the black circles represent outliers. Asterisks indicate statistical significance, as determined by two-tailed Student’s *t* test: ***P < 0.001.

Galactinol may be hydrolyzed to myo-inositol and galactose to provide alternative energy substrates in the dark. Our GWAS results identified SNPs associated with galactinol levels (Cluster 1) in the 3 and 6 days datasets with the lead SNP (with lowest *P*-value) being Chr5:14542999 (Figure 3A; Supplemental Figure S5, A and B). The linkage disequilibrium (LD) plot determined that the overlap between associated loci from the different datasets consists of nine genes, with the lead SNP Chr5:14542999 located in the ninth intron of At5g36890, which encodes BGLU42, involved in sugar catabolism. As galactinol content continually decreased during exposure to darkness (Figure 1B), we focused on *BGLU42* as the putative causal gene of this association. We obtained three independent T-DNA insertion lines to analyze the function of *BGLU42* in galactinol catabolism. The T-DNA of two lines (*bglu42-1* and *bglu42-3*) were inserted in the promoter region and induced the expression of *BGLU42*, while the T-DNA in the *bglu42-2* line was located in the twelfth exon and decreased *BGLU42* expression (Figure 3, B–C). Metabolomic analysis indicated that the *bglu42-1* and *bglu42-3* lines exhibit lower galactinol levels, whereas the *bglu42-2* line accumulated more galactinol compared to Col-0 controls. Similarly, the *bglu42-1* and *bglu42-3* lines displayed higher Chl content and *Fv/Fm* than Col-0, whereas *bglu42-2* mutant plants showed the opposite pattern (Figure 3, C–E; Supplemental Figure S5C). These results confirmed that galactinol catabolism may represent an additional C source for plant survival under darkness, with *BGLU42* potentially being a necessary gene involved in this process. As galactinol is hydrolyzed by α-galactosidases, we tested if BGLU42 possesses α-galactosidase activity; however, Arabidopsis *BGLU42* failed to complement an *Escherichia* *coli* α-galactosidase loss-of-function mutant (*ΔmelA*) (Supplemental Figure S5D). These results suggest that BGLU42 may be involved indirectly in galactinol metabolism.

The trehalose pathway acts as a sensor of energy status and C starvation; in addition, the hydrolysis of trehalose can supply two glucose moieties that can be used for energy production (Henry et al., 2014; Garapati et al., 2015). The continually decreasing pattern of trehalose contents in this study further indicated its important role in energy metabolism upon extended darkness (Cluster 2, Figure 1B; Supplemental Data Set S6). The LD plot revealed that the lead SNPs for the 0, 0–3, and 0–6 days datasets are identical (SNP Chr4:12487066), while the lead SNP in the 6 days dataset was SNP Chr4:12491454. The overlap between associated loci across the different datasets contained 10 genes (Supplemental Figure S6, A and B). Among them, At4g24040 (*TREHALASE 1* [*TRE1*]) caught our attention as its encoded protein can directly hydrolyze trehalose, and its expression is induced during darkness (Lin and Wu, 2004; Van Houtte et al., 2013; Yu et al., 2016; Liu et al., 2017). To identify the potential causative SNP in *TRE1*, we analyzed the 175 SNPs whose frequency was >1% in the *TRE1* region around the lead SNP. However, among the 175 SNPs, no SNPs were in high LD (*r^2^* > 0.7) with the two lead SNPs. Furthermore, to ask whether these two lead SNPs affect *TRE1* expression, we randomly selected 38 Arabidopsis accessions, of which 27 carried the T allele and 11 harbored the A allele at Chr4:12487066, and 32 accessions (six accessions) with the C allele (T allele) at Chr4:12491454. Accessions with the A allele at Chr4:12487066 or the T allele at Chr4:12491454 exhibited significantly higher trehalose content than those with the T allele and C allele at 0 and 6 days into darkness treatment (*P* < 0.001 and *P* < 0.05, respectively), while there was no significant difference in expression between the other genotypes (Supplemental Figure S6C). Taken together, *TRE1* played an important role in the observed variation in trehalose content, although the exact mechanism is currently unknown.

Besides the two SNPs associated with the sugars and sugar alcohols whose levels decrease, we identified one SNP associated with glycine levels (Cluster 1) on chromosome 3 in the 6 and 0–6 days datasets (Figure 3F; Supplemental Figure S7, A and B). The LD plot indicated that the lead SNP Chr3:20314932 is located in the first exon of At3g54830 (*AVT1B*), which encodes an amino acid vacuolar transmembrane transporter. To determine whether *AVT1B* is the causal gene, we obtained two independent knockdown mutants (*avt1b-1* and *avt1b-2*). Metabolite profiling indicated that glycine content is significantly lower in these two mutants relative to Col-0 plants grown in normal conditions (Figure 3, G–H). Moreover, the expression of *AVT1B* was strongly repressed by exposure to darkness (Supplemental Figure S7C). It is important to note that the glycine content of the two mutants was already extremely low under normal conditions and only decreased slightly more after transfer to extended darkness, providing a rationale for the absence of significant difference in glycine contents between Col-0 and the mutants after 6 days in darkness (Figure 3G). Taken together, these results suggest that *AVT1B* is an important negative regulator of glycine degradation. Furthermore, we determined that among the 180 SNPs (with a frequency exceeding 1%) mapping to the *AVT1B* region, 60 SNPs were in strong LD with the lead SNP Chr3:20314932 (*r^2^* > 0.7) (Figure 3I; Supplemental Table S1) and 31 were located in the *AVT1B* gene body region. Only two SNPs resulted in amino acid changes relative to the Col-0 reference sequence (blue SNPs in Figure 3I): Asp-30-Glu (Chr3:20314941, GWAS *P*-value = 1.06 × 10^−7^) and Thr-126-Ser (Chr3:20314570, GWAS *P*-value = 6.49 × 10^−7^). The remaining 29 SNPs located in the promoter region and may affect *AVT1B* expression. To test this possibility, we randomly selected 35 Arabidopsis accessions for expression analysis. Accessions with the T allele (*N* = 13) at Chr3:20314932 accumulated more glycine and expressed *AVT1B* to higher levels than those with the A allele (*N* = 22) only after 6 days of darkness treatment (Figure 3J). This result indicated that the haplotype linked to the T allele can increase *AVT1B* expression following the onset of darkness. As transmembrane domains are an important part of integral membrane transporters such as AVT1B and directly affect their function (Ortiz-Lopez et al., 2000), we next produced AVT1B hydrophobicity plots at the TMHMM server (Moller et al., 2001): AVT1B contained six transmembrane domains, but no amino acid substitutions (Asp-30-Glu and Thr-126-Ser) located within the transmembrane domains, suggesting they may not affect protein function (Supplemental Figure S7D). In summary, the 29 SNPs in the promoter region that were in strong LD with the lead SNP Chr3:20314932 appear to affect *AVT1B* expression and thereby glycine degradation under extended darkness.

### Genetic regulation of ornithine levels

Besides the decreasing metabolite patterns that result from energy supplementation under C starvation caused by extended darkness, high levels of N are likely rapidly recycled and (re)assimilated by the glutamine synthetase/glutamate synthase and urea cycles as the result of protein degradation (Kennedy et al., 2019). The metabolites glutamine, glutamate, and asparagine (related to the glutamine synthetase/glutamate synthase cycle), as well as ornithine, arginine, and urea (related to the urea cycle) belonged to Cluster 4, as they continually increased during exposure to darkness. Moreover, several organic acids and PCs shared the same pattern and the GWAS analysis identified some interesting associations, such as that between SNP Chr3:19978371 at the At3g53910 (*MALATE DEHYDROGENASE-LIKE PROTEIN*) locus and isocitric acid levels (*P*-value = 4.25 × 10^−7^) in the 3 days dataset; SNP Chr1:1935205 close to At1g06290 (*ACYL-COA OXIDASE 3* [*ACX3*]) and At1g06310 (*ACX6*) was associated with PC 32:3 (1) levels (*P*-value = 7.57 × 10^−7^) in the 6 days dataset (Figure 2A; Supplemental Data Set S2).

**Figure 4:**
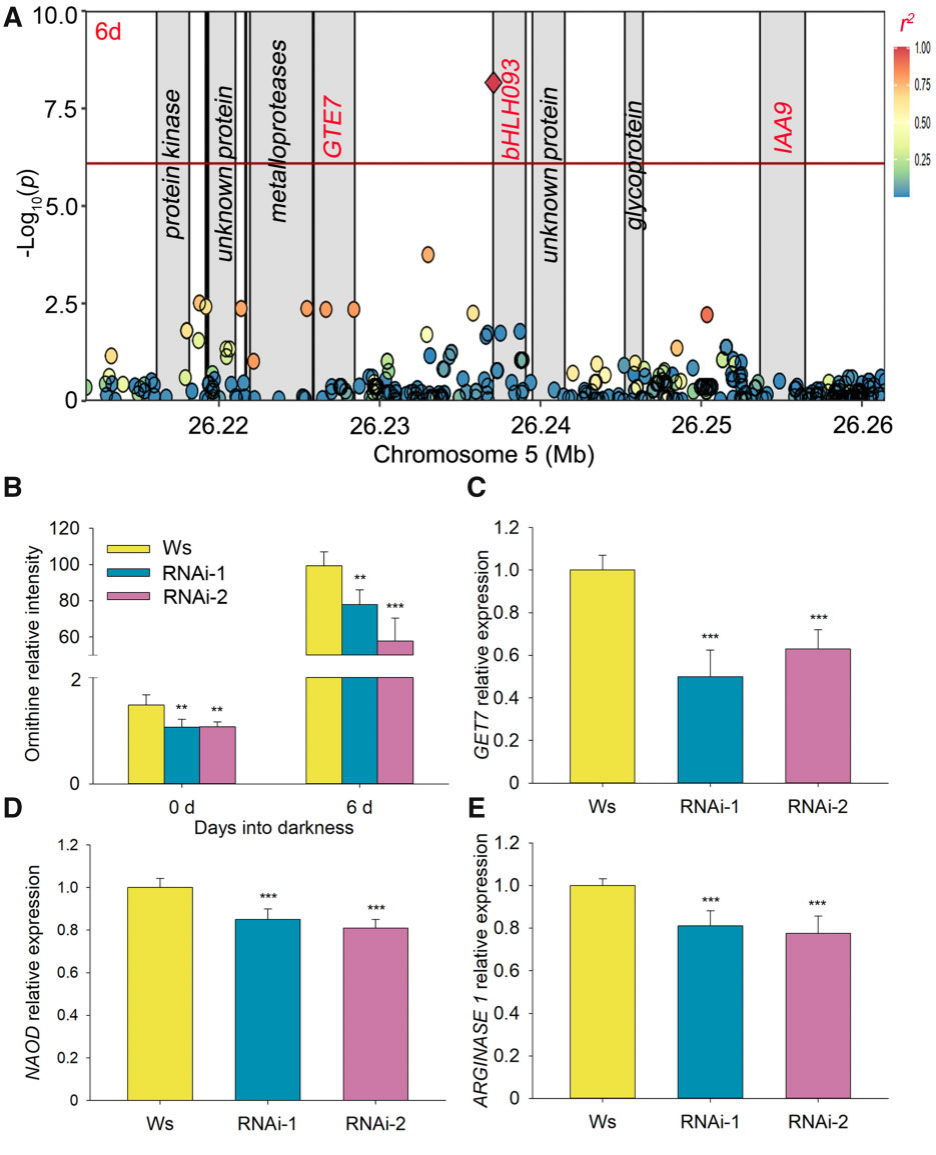
Association between *GTE7* and ornithine variation under extended darkness. A, LD plot based on imputed 1.2 M SNP data for the associations with ornithine levels for the 6 days dataset. The *x*- and *y*-axes are as in Figure 3A. Each gray block denotes a gene and each circle indicates an SNP, with the lead SNP (with lowest *P*-value) shown as a red diamond. The color of each circle reflects the *r^2^* value with the lead SNP.B–E, Phenotypes of *GTE7* RNAi*-*1 and RNAi-2 lines under extended darkness. B, Relative *GTE7* expression levels in Ws and *GTE7* RNAi lines, as determined by RT-qPCR (C–E) Ornithine contents and relative expression levels of the ornithine biosynthesis genes *NAOD* (D) and *ARGINASE 1* (E) in Ws and *GTE7* RNAi lines. Data are shown as means ± sd (*n* = 6 biological replicates). Asterisks indicate statistically significant differences relative to Ws, as determined by two-tailed Student’s *t* test: **P < 0.01; ***P < 0.001.

Among these associations, we identified one locus associated with ornithine levels on chromosome 5 in the 6 and 0–6 days datasets (Figure 4A; Supplemental Figure S8, A and B). The lead SNP Chr5:26237084 was located in the 5′-untranslated region (UTR) of At5g65640, which encodes the basic helix–loop–helix (bHLH) transcription factor bHLH93 (Figure 4A). As transcriptional regulators may affect the expression of genes associated with ornithine metabolism, we selected the three transcriptional regulators *bHLH093*, *GLOBAL TRANSCRIPTION FACTOR GROUP E7* (*GTE7*), and *INDOLE-3-ACETIC ACID INDUCIBLE 9* (*IAA9*), which are all located within a 4-kb window on chromosome 5, to analyze their effects on ornithine content variation. For validation experiments, we obtained one T-DNA insertion line for *bHLH093*, two independent RNA interference (RNAi) lines for *GTE7* and two independent T-DNA insertion lines for *IAA9*. All genotypes were grown alongside their respective wild-types (Col-0 for the T-DNA lines; Ws for the RNAi lines). All mutants expressed their target genes at significantly lower levels than their wild-type, but only the *GTE7* RNAi lines displayed a significantly lower ornithine content compared to their wild-type parent (Ws) under both normal and extended darkness conditions (Figure 4, B and C; Supplemental Figure S8C). Moreover, the expression of ornithine biosynthesis genes (such as *N(2)-ACETYLORNITHINE DEACETYLASE* [*NAOD*, At4g17830] and *ARGINASE 1*, At4g08900) were significantly downregulated by about 20% relative to Ws in the *GTE7* RNAi lines (*P* < 0.001; Figure 4, D and E). These results indicated that *GTE7*, which is repressed under dark stress (Lin and Wu, 2004; Yu et al., 2016), affects the expression of genes associated with ornithine metabolism and induces variation in ornithine contents under extended darkness.

### Genetic regulation of tyrosine and threonine levels

Following the global degradation of proteins upon extended darkness, the contents of several amino acids such as BCAAs, tyrosine, lysine, and threonine rapidly increased during early darkness treatment; with the further extension of darkness, these metabolites may have acted as important alternative respiration substrates and become degraded to supply energy for plant survival. These metabolites grouped into Cluster 5, with a peak 3 days into darkness. In addition to these metabolites, TAGs, β-alanine, nicotinic acid, and some PCs also belonged in Cluster 5. As the loci associated with TAG 54:9, β-alanine levels as well as the involvement of the 3-hydroxyisobutyryl-CoA hydrolase CHY4 on leucine degradation have been previously reported (Wu et al., 2016; Gipson et al., 2017; Luzarowska et al., 2020; Supplemental Figure S4; Supplemental Data Set S2) and corroborate the accuracy of our present analysis, we focused on two loci associated with tyrosine and threonine levels to illustrate their detailed genetic regulation.

**Figure 5:**
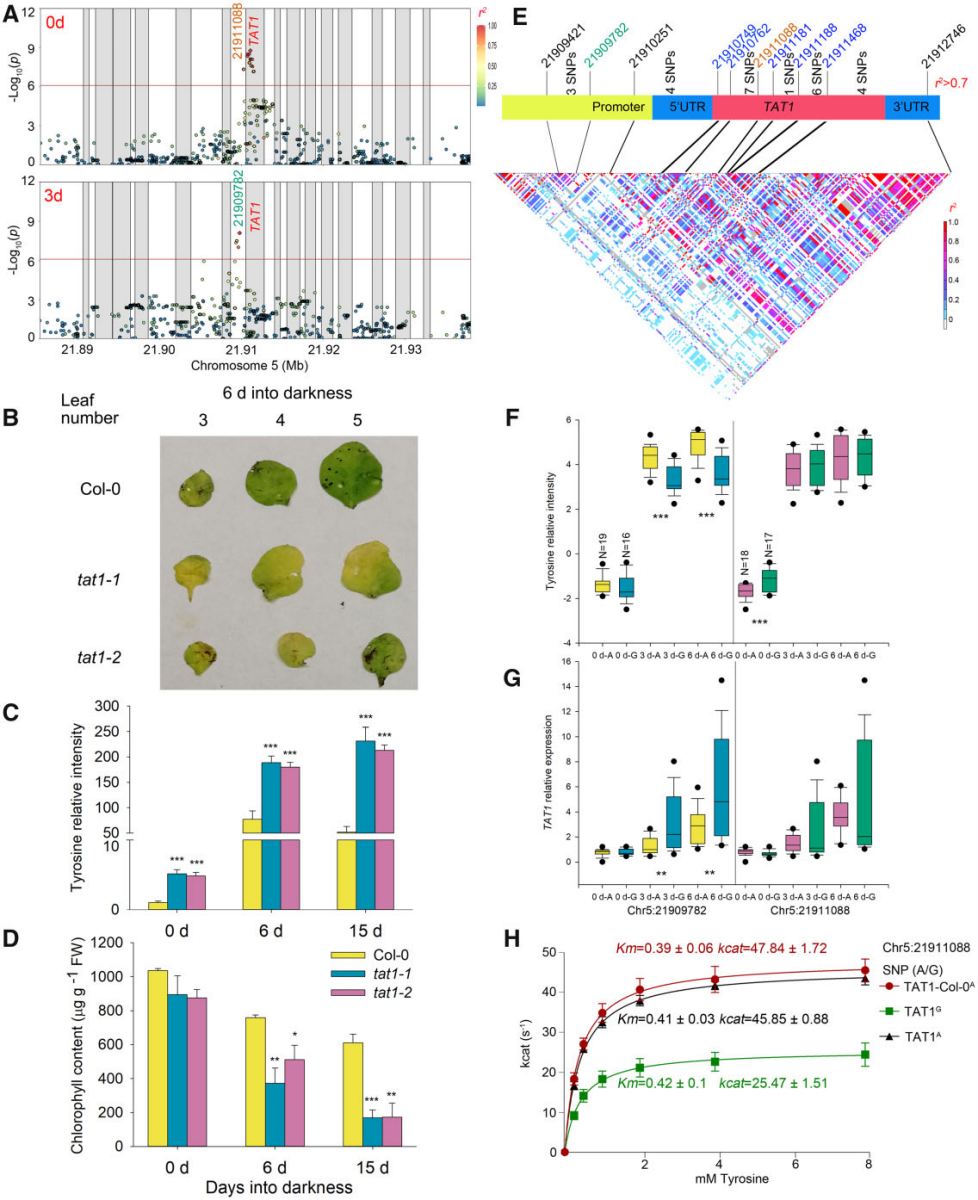
Distinct genetic mechanisms by which *TAT1* affect tyrosine contents in Arabidopsis accessions under normal and extended darkness conditions. A, LD plot based on the imputed 1.2 M SNP data for the associations with tyrosine content for the 0 and 3 days datasets. The *x*- and *y*-axes are as in Figure 3A. Each gray block denotes a gene and each circle indicates an SNP, with the lead SNP (with lowest *P*-value) shown as a red diamond. The color of each circle reflects the *r^2^* value with the lead SNP. B–D, Phenotypes of *tat1-1* and *tat1-2* mutants during extended darkness. B, Dark-induced senescence of Col-0 *tat1-1* and *tat1-2* mutants. Tyrosine (C) and Chl (D) content of Col-0, *tat1-1*, and *tat1-2* mutants at 0 days, or after 6 or 15 days into darkness. Data are shown as means ± sd (*n *= 6 biological replicates for (C), *n* = 3 for (D)). Asterisks indicate statistically significant differences relative to Col-0, as determined by two-tailed Student’s *t* test: *P < 0.05; **P < 0.01; ***P < 0.001. E, LD plot based on the SNPs around the *TAT1* region from the 1,001 Arabidopsis genomes database. The lead SNPs for 0 days and dark-related datasets are shown in purple and red, respectively. The SNPs located within the gene body region that change TAT1 protein sequence are shown in blue. F and G, Tyrosine content (F) and relative *TAT1* expression levels (G) of randomly selected accessions. The number of accessions with each genotype is given for the 0 days phenotypic values. The boxplots represent the interquartile range, the solid horizontal line represents the median, the whiskers represent 1.5× interquartile range, and the black circles represent outliers. Asterisks indicate statistical significance, as determined by two-tailed Student’s *t* test: **P < 0.01; ***P < 0.001. H, Enzymatic activity from the two TAT1 proteins based on SNP-Chr5:21911088 (TAT1^A^ and TAT1^G^). The Col-0 *TAT1* cDNA carries the A allele and was cloned as a positive control. The values are the mean of two independent assays of both two independent purifications (*n* = 2). Error bars indicate sd.

Our GWAS results indicated that tyrosine is a key metabolite during dark-induced senescence. Notably, we also identified SNPs associated with tyrosine levels on chromosome 5 in the 0 days dataset; importantly, the lead SNPs were different from those obtained with dark-related datasets, although they mapped to the same locus. Indeed, the lead SNP for the dark-related dataset was Chr5:21909782, while that for the 0 days dataset was Chr5:21911088, located in the promoter and first intron of *TAT1* (At5g53970), respectively (Figure 5A; Supplemental Figure S9; Supplemental Data Set S2). The SNP location, gene function, and significant expression induction of this gene by darkness indicated that *TAT1* is the most promising candidate gene related to tyrosine levels in both normal and extended darkness conditions (Lin and Wu, 2004; Yu et al., 2016; Liu et al., 2017). To analyze the function of *TAT1*, we measured the metabolite levels of two independent loss-of-function T-DNA insertion lines, *tat1-1* and *tat1-2* (Wang et al., 2019). Tyrosine contents significantly increased in Col-0 plants after transfer to darkness and reached a peak after 6 days, followed by a decrease by 15 days. In contrast, the *tat1-1* and *tat1-2* mutants already accumulated higher tyrosine under normal conditions, but tyrosine levels further rose upon transfer to extended darkness, even after 15 days (Figure 5, B and C). This finding thus confirmed the tyrosine degradation function of TAT1 under both normal and extended darkness conditions. Given that the degradation of tyrosine is an important contributor of respiratory substrates during dark-induced senescence, the low Chl content and *Fv/Fm* of the two mutants further indicated that *TAT1* is involved in tyrosine catabolism and aids plant survival under darkness (Figure 5D; Supplemental Figure S9C).

The LD analysis indicated that Chr5:21909782 significant in the dark-related datasets and Chr5:21911088 for the 0 days dataset are in weak LD (*r^2^* = 0.23), while four SNPs in the *TAT1* promoter region were in strong LD with the lead SNP Chr5:21909782 (*r^2^* > 0.7). The remaining 29 SNPs in the *TAT1* promoter and gene body region were in strong LD with the lead SNP Chr5:21911088 (*r^2^* > 0.7) (Figure 5E; Supplemental Table S2). Among the 29 SNPs, 5 SNPs altered the TAT1 protein sequence (blue SNPs in Figure 5E). To further investigate the causal genetic differences of the lead SNPs, we randomly selected 35 Arabidopsis accessions, of which 19 carried the A allele at Chr5:21909782 (and the remaining 16 the G allele) and 18 accessions harbored the A allele at Chr5:21911088 (and 17 accessions the G allele). For Chr5:21909782, accessions harboring the G allele accumulated lower tyrosine contents and exhibited higher *TAT1* expression compared to those with the A allele following 3 and 6 days into darkness. For Chr5:21911088, accessions carrying the A allele exhibited lower tyrosine content relative to those with the G allele under normal conditions only and did not display altered *TAT1* expression at any time point (Figure 5, F and G). To test the effects of lead SNPs on protein activity, we cloned versions of the *TAT1* coding sequence with either the A allele (from accessions CAM-61 and Col-0) or the G allele (from accession G-1) at Chr5:21911088. Enzyme kinetic analyses of these different TAT1 proteins indicated that the A allele TAT1^A^ shows a higher tyrosine degradation activity than the G allele TAT1^G^, although both proteins exhibited the same stability (Figure 5H; Supplemental Figure S9D). In summary, these results discovered at least two different mechanisms for the regulation of *TAT1* and thus tyrosine levels under normal and darkness conditions. Under normal condition, the five SNPs in high LD with SNP Chr5:21911088 resulted in different TAT1 enzymatic activity. Under extended darkness conditions, the lead SNP (Chr5:21909782) and four other promoter SNPs in strong LD affected *TAT1* gene expression, leading to variation in tyrosine levels under extended darkness.

**Figure 6:**
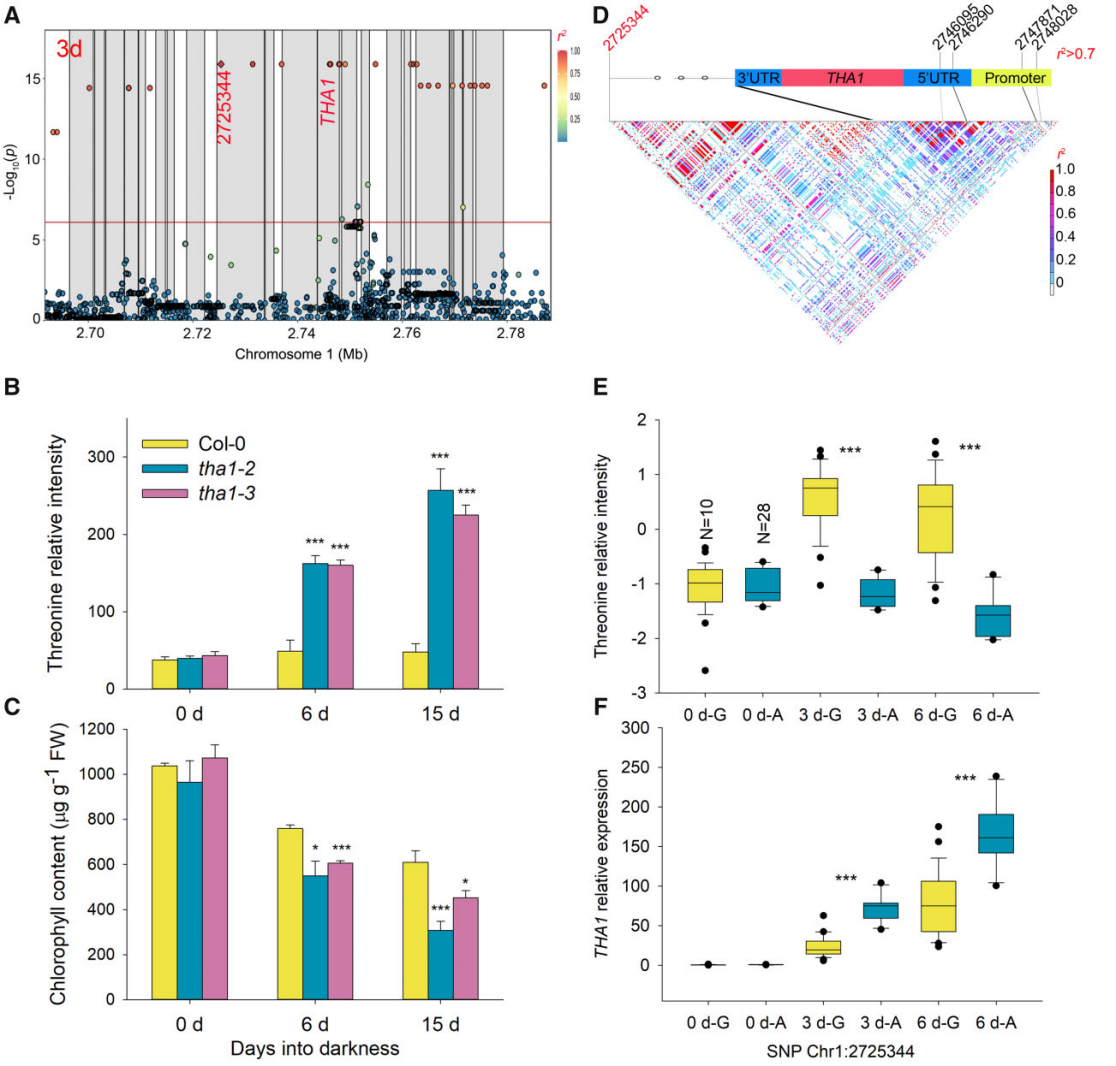
A possible genetic mechanism by which *THA1* affects threonine content in Arabidopsis accessions upon extended darkness. A, LD plot based on the imputed 1.2 M SNP data for the associations with threonine levels for the 3 days dataset. The *x*- and *y*-axes are as in Figure 3A. Each gray block denotes a gene and each circle indicates an SNP, with the lead SNP (with lowest *P*-value) shown as a red diamond. The color of each circle reflects the *r^2^* value with the lead SNP. B and C, Phenotype of Col-0, *tha1-2*, and *tha1-3* mutants in extended darkness. Threonine (B) and Chl (C) content in Col-0, *tha1-2*, and *tha1-3* mutants at 0 days, or after 6 and 15 days in extended darkness. Data are shown as means ± sd (*n* = 6 biological replicates for (B), *n* = 3 for (C)). Asterisks indicate statistically significant differences relative to Col-0, as determined by two-tailed Student’s *t* test: *P < 0.05; ***P < 0.001. D, LD plot based on the SNPs of the lead SNP (Chr1: 2725344) and around the *THA1* gene from the 1001 Arabidopsis genomes database. The lead SNP is shown in red. E and F, Threonine content (E) and relative *THA1* expression levels (F) of randomly selected accessions. The number of accessions with each genotype is given for the 0 days phenotypic values. The boxplots represent the interquartile range, the solid horizontal line represents the median, the whiskers represent 1.5× interquartile range, and the black circles represent outliers. Asterisks indicate statistical significance, as determined by two-tailed Student’s *t* test: ***P < 0.001.

Given its similar behavior in response to extended darkness (Cluster 5, Figure 1B; Supplemental Data Set S6) and proximity to representative alternative respiratory substrates, such as BCAAs and tyrosine in the metabolite network (Supplemental Figure S3), the same lead SNP, Chr1:2725344, was associated with threonine levels in the dark-related datasets (3, 6, 0–3, and 0–6 days datasets) (Figure 6A; Supplemental Figure S10A). Around this lead SNP, *THA1* (At1g08630) was the only gene related to threonine metabolism. We validated its function in threonine metabolism by phenotyping two independent mutants (*tha1-2* and *tha1-3*) under extended darkness (Figure 6B; Supplemental Figure S10, B–D). The threonine contents of leaves were comparable between the wild-type plants and the two mutants under normal conditions, which was in agreement with the results of a previous study (Joshi et al., 2006). After 6 and 15 days of darkness treatment, the threonine contents of the two mutants were significantly higher compared to Col-0 (*P* < 0.001; Figure 6B). Moreover, *THA1* expression after 6 or 15 days into darkness was 169- and 297-fold higher, respectively, compared to Col-0 in normal conditions (Supplemental Figure S10E; Lin and Wu, 2004; Yu et al., 2016; Liu et al., 2017). These results indicated that *THA1* plays a predominant role in regulating threonine content under extended darkness, likely explaining why this locus was only identified in dark-related datasets. Furthermore, the two *tha1* loss-of-function mutants exhibited low Chl content and *Fv/Fm* values when transferred to extended darkness (Figure 6C; Supplemental Figure S10D). These results indicated that threonine catabolism, in addition to that of aromatic amino acids and BCAAs, plays an important role in plant survival under darkness and that *THA1* is a necessary gene involved in this process. Moreover, among the SNPs in the *THA1* region, four SNPs in the promoter and 5′-UTR region were in strong LD with the lead SNP Chr1:2725344 (*r^2^* > 0.7) (Figure 6D; Supplemental Table S3). We randomly selected 38 Arabidopsis accessions carrying either allele at Chr1:2725344: 10 accessions with the G allele and 28 accessions with the A allele. Accessions harboring the G allele had significantly higher threonine content and lower *THA1* expression than those with the A allele at 3 and 6 days into darkness treatment, while we observed no significant difference for threonine content or *THA1* expression at 0 days (*P* < 0.001; Figure 6, E and F). This result indicated that the haplotype with the A allele might increase *THA1* expression levels and decrease threonine contents under extended darkness.

## Discussion

During dark-induced senescence, metabolism is dramatically reprogrammed to lengthen the lifespan of the plant by switching from anabolic to catabolic processes and recycling and remobilizing C and N sources for use in sink tissues. However, previous research on this topic largely focused on individual genes or pathways by characterizing their mutants or one specific stage. To gain a broader understanding of the events underlying dark-induced senescence, we performed a time-resolved GWAS of *Fv/Fm*, Chl, primary metabolites, and lipid contents at 0, 3, and 6 days into darkness. Given that chloroplasts are the main source for N recycling, the degradation of chloroplasts is vital for plant survival during dark-induced senescence and can be evaluated by measuring the *Fv/Fm* and Chl content. Previous work using 503 diverse maize (*Zea mays*) inbred lines exhibiting substantial phenotypic variation in *Fv/Fm* ranging from 0 to 0.80 and with high heritability (0.37–0.73) identified several genes involved in leaf senescence (Sekhon et al., 2019). However, in Arabidopsis, the smaller range (0.40–0.70) and much lower heritability (0.15–0.25) of *Fv/Fm* values under normal and cold conditions strongly reduced the power of GWAS to detect genomic regions showing significant association, forcing an adjustment of the genome-wide analysis threshold to 1 × 10^−4^ (using 215,000 SNPs in GWAS) prior to finding some PSII-associated proteins (van Rooijen et al., 2015). In this study, the results were in alignment with previous Arabidopsis research in that the range of values seen for *Fv/Fm* was 0.723–0.764 (0 days), 0.591–0.654 (3 days), and 0.324–0.654 (6 days) and the CV is 0.008 (0 days), 0.018 (3 days), and 0.068 (6 days) at the different time-points (Supplemental Data Sets S1 and S5). Moreover, the broad-sense heritability of *Fv/Fm* among the three conditions was only 0.030 (Supplemental Data Set S5). It is therefore not unexpected that just one SNP exceeds the genome-wide threshold for this environmentally sensitive trait (Supplemental Data Set S2). Moreover, Chl content also exhibited low variation and heritability, with only one association revealed by GWAS (Supplemental Data Sets S1 and S5). The low broad-sense heritability indicated that *Fv/Fm* values and Chl contents may be strongly influenced by environmental effects or limited by the population size in this study. In the future, special treatments (such as different growth light intensities or guazatine treatment) and a larger population size will likely be needed for further analysis and to mine the genetic contribution to these phenotypes (van Rooijen et al., 2015; Atanasov et al., 2016; van Rooijen et al., 2017).

**Figure 7:**
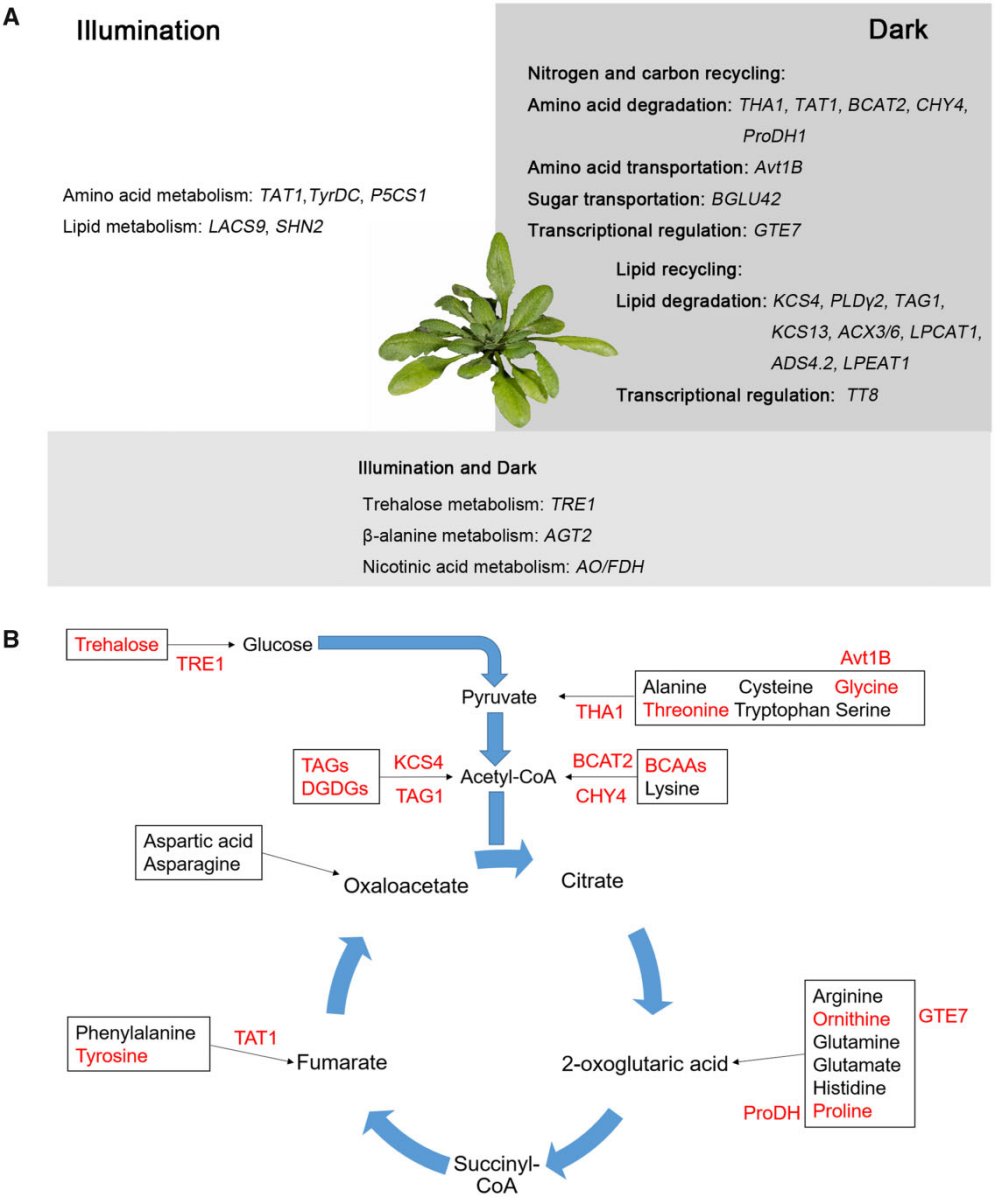
Summarizing the genetics landscape of Arabidopsis primary and lipid metabolism under light and dark conditions. A, Under illumination, the variation in tyrosine levels is attributed to the variation in TAT1 enzyme activity resulting from five amino acid changes. *TAT1*, *TyrDC*, *P5CS1*, and *SHN2*contribute to the variation of the related primary and lipid metabolites. Under dark conditions, metabolite recycling becomes activated and the different expression levels of *TAT1*, *THA1*, and *AVT1B* affect tyrosine and threonine degradation as well as glycine transport during recycling. In addition, *BCAT2*, *CHY4*, *ProDH1*, *BGLU42*, and *GTE7* also modulate the variation of N and C recycling. As an important source of energy, variation in lipid recycling is affected by *KCS4*, *PLDγ2*, *TAG1*, *KCS13*, *ACX3/6*, *LPCAT1*, *ADS4.2*, *LPEAT1*, *PLDALPHA2*. Besides these genes, *TRE1*, *AGT2*, and *AO/FDH* are responsible for variation in trehalose, β-alanine, and nicotine acid metabolism under light or dark conditions, respectively. These changes, at least partially, explain the metabolic shifts that take place during dark-induced senescence. B, Glycolysis and the TCA cycle are the most important pathways that supply energy for survival under stress imposed by extended darkness. During darkness, sugars such as trehalose can produce glucose under the action of TRE1. Lipid metabolites such as TAGs and DAGs can be degraded by KCS4 and TAG1. Moreover, different amino acids (threonine, BCAAs, proline, and tyrosine) act as alternative respiratory substrates to produce pyruvate, acetyl-CoA, 2-oxoglutaric acid, and fumarate through the regulation of THA1, BCAT2, CHY4, ProDH1, and TAT1. Glycine and ornithine metabolism under darkness-induced stress is regulated by AVT1B and GTE7.

Despite the somewhat disappointing outcome mentioned above, we went ahead and assessed metabolite levels under all conditions, as one of our aims was to understand what orchestrates the metabolic status on dark-induced senescence. In fact, we posit that for this purpose, the lack of major effects at the level of *Fv/Fm* values and Chl contents may even be an advantage, as the genetic architecture of metabolite accumulation is less likely to be occluded by pleiotropic effects. While this comment may seem excessive from the measurement of these two traits alone, it is important to note that we did not observe any obvious physiological differences in how accessions responded to darkness. The primary effect of darkness is the cessation of photosynthesis and the shift from an autotrophic to a heterotrophic status, leading to the degradation and recycling of proteins, lipids, and sugars as a means of plant survival (Figure 7A). Given that glycolysis and the TCA cycle are the most important pathways to supply energy for survival under darkness, different alternative respiratory substrates can be degraded to metabolites to support these two pathways (such as pyruvate, acetyl-CoA, 2-oxoglutaric acid, and fumarate) under the genetic regulation of the associated enzymes (Figure 7B). In this study, we identified six patterns of metabolite shifts; the metabolites directly involved in energy generation (sugars, sugar alcohols, and the organic acids of the TCA cycle) decreased significantly when the darkness was initiated (Clusters 1–3 in Figure 1B; Supplemental Data Set S6). Moreover, given the high level of amino acid degradation in darkness, N is likely rapidly recycled through aminotransferase reactions to form glutamate and (re)assimilated by the glutamine synthetase/glutamate synthase cycle and the urea cycle (Lam et al., 1998; Kennedy et al., 2019). The fact that glutamine, glutamate, asparagine, ornithine, and arginine continued to rise further confirmed the high rate of N recycling and remobilization during darkness (Cluster 4 in Figure 1B; Supplemental Data Set S6). As another important connection between N and C metabolism, the stable increase of gamma-aminobutyric acid further indicated the use of alternative respiratory substrates during this process (Studart-Guimarães et al., 2007) (Cluster 4 in Figure 1B; Supplemental Data Set S6). Furthermore, following initially increasing levels, presumably caused by protein and lipid degradation during early darkness, the levels of BCAAs, tyrosine, and lysine decreased significantly upon extended darkness (Araujo et al., 2010; Peng et al., 2015; Fan et al., 2017; Wang et al., 2019) (Cluster 5 in Figure 1B; Supplemental Data Set S6). Together, these results indicated that the metabolite shifts seen in Arabidopsis are under highly coordinated regulation to counteract the C starvation caused by extended darkness. The continually decreasing metabolites (Clusters 1–3, such as trehalose, galactinol, and glycine) may act as early-responding metabolites for recycling, while those increasing and then decreasing (Cluster 5, such as tyrosine, threonine, and BCAAs) may be late-responding metabolites that act as alternative respiration substrates to supply energy under extended darkness.

**Figure:**
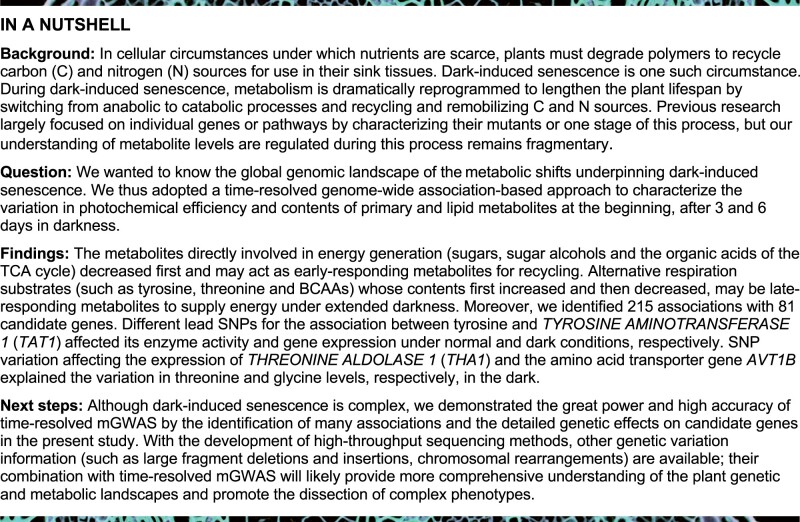


### The genes underlying the recycling and remobilization of metabolites

Metabolite recycling and remobilization under darkness stress were regulated at different levels, such as the enzyme catalyzing the macronutrient degradation (for proteins, amino acids, sugars, or lipids), metabolite transport, and transcriptional regulators of the encoding genes. Interestingly, based on the GWAS of the different datasets, the analysis of the metabolites exhibiting different shift patterns identified several genes belonging to individual regulatory mechanisms. Although the levels of amino acids are low in the cell, their conversion products can not only directly enter the TCA cycle to produce ATP and electrons, but also provide electrons to the electron transport chain via the electron transfer flavoprotein complex (Araújo et al., 2011). In theory, the energy yield of tyrosine is 34 ATP molecules, which is comparable to the oxidation of glucose as substrate (32 ATPs; Hildebrandt et al., 2015). In this study, *TAT1* exhibited strong associations with tyrosine levels not only under normal conditions but also in the dark-related GWAS results (Figure 5A; Supplemental Figure S9A). Moreover, the *tat1-1* and *tat1-2* T-DNA insertion mutants accumulated higher tyrosine contents under normal conditions that further and continually increased upon extended darkness (Figure 5, B–D). These observations are in agreement with the function of the TAT1 enzyme in supplying electrons to cellular respiration and providing TCA cycle substrates (e.g. fumarate), which plays an important role in the cellular energy balance under stress brought upon by extended darkness (Wang et al., 2016, 2019). Interestingly, *BCAT2* only displayed a significant association with BCAA levels in the 6 and 0–6 days datasets based on the 200 K SNPs Affymetrix Chip data (Supplemental Figure S2; Supplemental Data Set S4). Furthermore, we observed no differences for BCAA contents in *bcat2-1* and *bcat2-2* T-DNA insertion mutants under normal conditions, although the mutants did exhibit significant differences in their BCAA contents and dark-induced senescence phenotype relative to wild-type plants following darkness. These results may be attributed to the low expression of *BCAT2* under normal conditions, and its dramatic induction upon darkness treatment (Supplemental Figure S2). This hypothesis is in line with a previous GWAS study concerning BCAA contents of Arabidopsis seeds (Angelovici et al., 2013). In addition, the gene encoding an important enzyme involved in the degradation of BCAAs, *CHY4*, also exhibited a strong association with leucine levels in dark-related datasets (Supplemental Figure S4; Supplemental Data Set S2; Gipson et al., 2017).

Besides tyrosine and BCAAs, our results also indicated that threonine levels follow similar changes during darkness, suggesting that threonine may act as a novel respiration substrate for energy supply under darkness (Figure 1B; Supplemental Figure S3). In the GWAS analysis, *THA1* was strongly associated with threonine levels in the dark-related datasets, while a previous study reported that *tha1* loss-of-function mutants only result in a rise of threonine contents in seeds, but no in seedlings (Joshi et al., 2006). These results indicated that *THA1* may act as a senescence-specific regulator of threonine contents during seedling senescence. The expression and loss-of-function mutants phenotype confirmed this hypothesis: the expression of *THA1* was induced several hundred-fold following transfer into extended darkness and the loss-of-function *tha1* mutants only accumulated high threonine levels following darkness and exhibited an accelerated senescence phenotype specifically in the dark (low Chl content and low *Fv/Fm*) (Figure 6, B and C; Supplemental Figure S10D). Besides these amino acids, the contents of galactinol continuously decreased in extended darkness, which may be explained by its removal by export or hydrolysis. In the 3 and 6 days datasets, *BGLU42* was highly associated with galactinol levels; *bglu42* T-DNA insertion mutants indicated that *BGLU42* acts as a negative regulator of galactinol contents and affects dark-induced senescence (Figure 3, A–E), as does its maize homolog (Sekhon et al., 2019). However, Arabidopsis *BGLU42* failed to complement an *E. coli* α-galactosidase loss-of-function mutant (Supplemental Figure S5D), suggesting that BGLU42 may not directly hydrolyze galactinol and may instead be involved in its metabolism or export via an indirect mechanism.

Membrane lipids are another alternative important C source for plant cells that are degraded to TAGs and subsequently degraded through β-oxidation to produce vital alternative respiration substrates (Avin-Wittenberg et al., 2015). In this study, consistent with data presented previously (Luzarowska et al., 2020), we discovered a very strong association between *3-KETOACYL-COA SYNTHASE 4* (*KCS4*) and unsaturated TAGs in the dark-related datasets, supporting the important role of *KCS4* in lipid recycling upon darkness (Supplemental Data Set S2). During recycling, transport of the compounds is also a crucial step for the transformation of metabolites. Under darkness, glycine levels continually decreased, potentially as a result of its conversion to serine in mitochondria (Figure 1B; Supplemental Data Set S6; Engel et al., 2007). Moreover, the 6 days GWAS indicated that *AVT1B* is highly associated with glycine levels; importantly, validation experiments showed that *AVT1B* acts as a positive regulator of glycine content (Figure 3, F–J). As the AVT1 subfamily function in vacuolar uptake (Russnak et al., 2001), we postulate that AVT1B acts as a vacuolar glycine transporter and can thus promote the storage of glycine.

In addition to the structural genes directly involved in metabolism and transport, transcriptional regulators participate in metabolite recycling. In the 6 and 0–6 days datasets, we observed the strong association between the gene encoding the bromodomain-containing transcriptional regulator GTE7 and ornithine levels. We confirmed the positive role of GTE7 on ornithine content with *GTE7* RNAi lines (Figure 4). Moreover, the expression of ornithine biosynthesis genes (such as *NAOD* and *ARGINASE 1*) in *GTE7* RNAi lines were significantly downregulated compared to their wild-type (Figure 4B). As bromodomain-containing proteins can specifically recognize acetylated lysine residues of histones and affect histone acetylation, chromatin remodeling, and the recruitment of other trans-factors for transcription (Josling et al., 2012), we postulate that GTE7 may affect the histone acetylation levels of ornithine metabolism genes and thus contribute to the variation in ornithine levels in the population. In addition to GTE7, several transcription factors have been reported to regulate lipid metabolism and in this study, we identified *SHN2* as exhibiting a high association with DAG 34:2 levels (Supplemental Figure S4; Supplemental Data Set S2), which was in agreement with its function in lipid metabolism (Aharoni et al., 2004). Moreover, in the GWAS results, we also found several autophagy genes (*APG9*), the trypsin inhibitor (*ATKTI1*), the ubiquitin protease (*UBP18*) whose presence is in agreement with their function in the metabolite recycling (Hanaoka et al., 2002; Li et al., 2008) (Supplemental Data Set S2).

## Conclusion

In this study, capitalizing on the high-intensity SNP information from the 1,001 Arabidopsis Genomes project, we analyzed the detailed genetic variation underlying 215 associations mapping to 81 genes and identified several causal SNPs located in different regions of candidate genes (Supplemental Data Set S2). Among them, 14 lead SNPs were in high LD with SNPs that can change the protein sequence encoded by the candidate genes, with another 45 lead SNPs that are in high LD with SNPs located in the near intergenic region, introns, or promoter regions and may thus affect the expression of the candidate genes. One of the most interesting findings concerns the association between tyrosine levels and *TAT1*, whose lead SNP affected TAT1 enzymatic activity and *TAT1* transcriptional regulation under normal and darkness conditions, respectively (Figure 5). Moreover, we attributed the variation of threonine and glycine levels to the differential expression of *THA1* and *AVT1B*, whose lead SNPs were also in high LD with the SNPs in their promoter regions (Figures 3, F–J and 6). Given that *TAT1* and *THA1* expression was significantly induced while that of *AVT1B* is repressed upon extended darkness, it additionally appears that changing gene expression may be an important mechanism by which plants adapt to stress imposed by darkness.

This study already allowed us to dramatically refine our understanding of the metabolic shifts taking place during dark-induced senescence. Beyond this particular stress, we believe we have additionally demonstrated the power of time-resolved mGWAS as an approach that represents a powerful approach to dissect complex phenotypes, despite the huge amount of work involved. Besides SNPs, structural variation such as large fragment deletions and insertions or chromosomal rearrangements also play an important role in plant evolution and agricultural traits (Alonge et al., 2020; Dominguez et al., 2020). In future computational studies using data acquired by high-throughput long-read sequencing strategy and obtaining population-scale SV information, time-resolved mGWAS approaches will likely provide more information concerning both the genetic and metabolic landscapes of plants.

A further important finding of this work is that we validated the strong link between *THA1*, *BGLU42*, *TAT1*, and *BCAT2* to dark-induced senescence, as knockout mutants in each gene displayed low Chl content and compromised photochemical efficiency following extended darkness (Supplemental Figure S11). These results thus offer insights into these traits in spite of the lack of clear associations with the natural variation underlying these genes. As such, our study confirms the value of reverse genetic intervention as a means of both validating candidate genes and evaluating function in a less subtle manner than offered by natural diversity. It thus seems likely that such approaches will prove useful for the temporally based dissection of complex composite traits.

## Materials and methods

### Plant materials and growth conditions

To maximize diversity and minimize redundancy and close family relatedness, a previously assembled natural diversity panel of *A.* *thaliana* accessions was used to study dark-induced senescence (Wu et al., 2016, 2018). The information on these accessions is listed in Supplemental Data Set S8. To obtain reliable data for the GWAS analysis, among the 288 accessions that germinated in the experiment, only 252 Arabidopsis accessions were included in the GWAS analysis, as they were harvested at all three time points during both seasons.

All T-DNA lines were obtained from the Nottingham Arabidopsis Stock Center. The information on the T-DNA lines used in this study is listed in Supplemental Table S4. The Primer Design Tool provided by the Salk Institute Genomic Analysis Laboratory (http://signal.salk.edu/tdnaprimers.2.html) was used to design the genotyping primers used to check T-DNA insertion and zygosity in the offspring. All primers are listed in Supplemental Table S5.

Arabidopsis seeds were sown directly on soil in 10-cm pots, and grown under a short-day (SD) photoperiod in a greenhouse (8-h light, 250 µE m^−2^ s^−1^, day/night temperature of 20°C/16°C and humidity 60%/75%). After 2 weeks, each accession was moved to an independent 6-cm pot for the three sample collecting time points at 0 and 3 days or 6 days after transfer into darkness. The seedlings were grown under SD conditions for 3 weeks. Pots were placed randomly to avoid block effects during growth; each tray contained one Col-0 plant to monitor the extent of spatial variation across trays. Based on the PCA conducted for metabolites using all accessions, Col-0 plants collected at the same time point clustered closely, indicating minor variation. At 35-days postgermination, one copy of all accessions was harvested before plants were transferred to darkness (0 days) by snap-freezing in liquid N. The remaining two copies of all accession plants were moved to darkness and were harvested after 3 and 6 days of extended darkness. At each harvest time, plants were harvested within 30 min at 10 am (GMT + 2). Owing to a large number of plants and the limited greenhouse space, plants for GWAS analysis were grown twice in autumn 2018 and spring 2019 as two independent biological replicates. During each season, each accession was represented by a single plant for each time point. In validation experiments with T-DNA insertion lines under extended darkness, all plants were harvested before transfer to darkness (0 days), or after 6 or 15 days of extended darkness. All samples were stored at –80°C prior to further analysis.

### Primary and lipid metabolites profiling by GC–MS and LC–MS

Extraction of metabolites and Chl was performed as described previously (Salem et al., 2016). The resulting polar phase (200 µL) was dried using a SpeedVac concentrator and resuspended in methoxyamine–hydrochloride (20 mg/mL in pyridin) solution and then derivatized with *N*-methyl-*N*-trimethylsilyltrifloracetamide. One microliter of the derivatized sample mixture was injected into the GC–MS system. The GC–MS system was coupled to a time-of-flight mass spectrometer (Pegasus III, Leco). The Golm Metabolome database was used for cross-referencing the mass spectra, and the relative metabolite contents were determined by normalizing the integrated areas of the characteristic fragment ion traces to the integrated area of ribitol (*m/z* 319).

Furthermore, 500 µL of the upper, lipid-containing phase, was dried in a SpeedVac concentrator and resuspended in 250 µL acetonitrile: 2-propanol (7:3, v/v) solution. Two microliters per sample were injected into a Waters Acquity ultra-performance LC system coupled with Fourier transform MS in positive ionization mode. The workflow included peak detection, retention time alignment, and removal of chemical noise. Isotopic peak detection from the MS data was performed as described in Luzarowska et al. (2020). Identified lipids were confirmed by manual verification of the chromatograms using Xcalibur (version 3.0, Thermo-Fisher, Bremen, Germany).

To eliminate extraction and batch effects for the final processed data and to ensure the accuracy of the analysis about the different metabolite intensity between time points, two pattern quality controls were included in the experiment. First, during metabolite extraction, one Ex-QC sample was added from the same pool material under normal conditions every 40 analyzed samples and followed the same analysis pipeline. Second, during metabolite profiling of all samples, an identical quality control was added to every 14 samples; every batch of 60 samples (containing four quality controls) were then normalized to the four quality controls, as described in Alseekh et al. (2018).

### Calculation of best linear unbiased predictions, heritability, genome-wide association mapping, and LD analysis

After normalization and log-transformation of the metabolite intensities, the genotype effect was estimated between the two seasons for each time point. A linear mixed model was fitted to each metabolite with the genotype and seasonal effects (both set as random). The best linear unbiased predictions (BLUPs) of random effects were calculated using the R package *lme4* (Bates et al., 2015). The summations of fixed effects (intercept) and the BLUPs for the genotype effect of each metabolite were used as the metabolite levels for each time point during GWAS analysis.

To estimate the genotype effect across the three time points, the time point effect (i.e. light and dark conditions) and the interactions with genotype were added to the linear mixed model as random effects. The metabolite levels across time points were estimated as above. The heritability ($H^{2}$) was estimated based on the variance components of genotype ($\sigma_{g}^{2}$), time point ($\sigma_{t}^{2}$), interaction ($\sigma_{i}^{2}$), season ($\sigma_{s}^{2}$), and residual error ($\sigma_{\varepsilon }^{2}$), using the equation:

$$H^{2}=\frac{\sigma_{g}^{2}}{\sigma_{P}^{2}}=\frac{\sigma_{g}^{2}}{\sigma_{g}^{2}+\sigma_{t}^{2}+\sigma_{i}^{2}/t+\sigma_{s}^{2}/(t\times s)+\sigma_{\varepsilon }^{2}/(t\times s)}$$

where $\sigma_{P}^{2}$ is the phenotypic variance, t is the number of time points and s is the number of the season.

These data were mapped to the genetic loci using the *rMVP* (A Memory-efficient, Visualization-enhanced, and Parallel-accelerated tool) R package (Yin et al., 2021). *rMVP* employs a mixed linear model containing fixed and random effects. In *rMVP*, the population structure was characterized by using the first three PCs (Q matrix) (Price et al., 2006) to incorporate this information with the VanRaden kinship matrix (VanRaden, 2008) as fixed and random effects, respectively (method = “MLM”), in agreement with previous work using the same population (Segura et al., 2012; Fusari et al., 2017; Wu et al., 2018). The imputed 1.2 M-SNP data based on the 1,001 Arabidopsis Genomes project filtered with cross-validation accuracy over 0.95 was used for mapping (Arouisse et al., 2020). As the Bonferroni threshold is too stringent for quantitative gene identification, the genome-wide threshold of significance in this study was set to 8.10 × 10^−7^ (1/*N*, with *N* = 1,235,164 for the number of SNPs used in GWAS), as widely used in other work based on the same Arabidopsis population and in other species (Lander and Kruglyak, 1995; Wen et al., 2014; Kooke et al., 2016; Wu et al., 2021). The resulting SNPs with *P*-values less than the threshold were assigned to the same group if their inter-genomic distance was ˂20 kb. Genes within the resulting groups were considered as candidate genes. For the LD analysis based on the 1,001 Arabidopsis genome database, the SNP information was imported into TASSEL5.0 and the squared allele–frequency correlations (*r*^2^) of each SNP were calculated (Bradbury et al., 2007). Moreover, to improve the detection power of true associated genes, multiple trait GWAS considered the three time points at the same time in the response of the linear mixed model. The multivariate models were implemented and solved using the R package *sommer* (Covarrubias-Pazaran, 2018). The GWAS results based on the multivariate models were similar to those based on individual time points, especially for strong associations. Therefore, the GWAS results based on individual time points were used in the main text; the detailed results of multivariate GWAS models are listed in Supplemental Data Set S3. Additionally, as the imputation of the SNPs excluded some SNPs (and their associations) from the 200K SNPs Affymetrix Chip data, GWAS based on Affymetrix Chip SNP data using the mixed linear model was also performed. The detailed results are provided as Supplemental Data Set S4.

### Correlation-based network analysis from time-resolved data

Pearson’s correlation coefficients and associated *P*-values were calculated via the function corAndPvalue from the WGCNA package (Langfelder and Horvath, 2008) using the metabolite levels at all three time points (0, 3, and 6 days). Metabolite–metabolite pairs were retained that exhibited significant connections (*P*_adj_*-*value < 0.05, with false discovery rate control using the Benjamini–Hochberg method) and coefficients *r* > 0.5. The network was obtained from the resulting adjacency matrix using graph from adjacency matrix in the igraph package (mode = “undirected”, weighted = TRUE, diag = FALSE) (Csardi and Nepusz, 2006). These results were imported into Cytoscape (version 3.6.1) to visualize the network (Shannon et al., 2003).

### Quantification of enzymatic activity for TAT1 proteins

The *TAT1* coding sequences from different genotypes were PCR amplified from accession CAM-61 (ecotype.66), Col-0 (C allele), and from accession G-1 (ecotype.7150) (T allele) using cDNAs prepared from total RNA extracted from rosette leaves for each genotype at SNP Chr5:21911088 and then inserted into pET-28a (Novagen, Madison, WI, USA). The same expression construct described in Wang et al. (2016) was used for Arabidopsis *TAT1* from Col-0 (At5g53970.1). Chemically competent Rosetta-2 (DE3) *E.* *coli* cells (Novagen, Madison, WI, USA) were transformed with each vector and selected on LB agar plates containing 50 µg/mL kanamycin. Colonies for each construct were picked, inoculated in 10 mL LB medium with 50 µg/mL kanamycin, and incubated overnight at 37°C with shaking at 200 rpm. Two milliliter of each culture was transferred to 100 mL of fresh LB medium and grown at 37°C with shaking at 200 rpm until optical density at 600 nm (OD_600_) reached ∼0.55. The temperature was then dropped to 30°C and isopropyl β-d-1-thiogalactopyranoside was added to 0.2 mM final concentration. After overnight incubation at 30°C with shaking at 200 rpm, cells were harvested by centrifugation at 3,824 *g* for 20 min at 4°C. The pellet was either stored at −80°C for later use or resuspended in 2 mL of lysis buffer containing 50 mM sodium phosphate (pH 8.0), 300 mM NaCl, 25 µM pyridoxal-5-phosphate (PLP), and 0.25 mg/mL lysozyme (Sigma-Aldrich, St Louis, MO, USA). After disrupting cells by three freeze–thaw cycles and sonication, the supernatant was obtained by centrifugation at 18,000*g* for 30 min at 4°C. His-tagged recombinant proteins were purified using nickel-conjugated magnetic beads (PureProteome, Millipore, Burlington, MA, USA) according to manufacturer’s protocol and further desalted by Zeba Spin Desalting Columns (Thermo Scientific, Waltham, MA, USA) into 100 mM HEPES buffer (pH 7.5) containing 25 µM PLP and 10% (v/v) glycerol. Recombinant proteins were separated by sodium dodecyl sulfate polyacrylamide gel electrophoresis (SDS-PAGE), and the gels were stained with Coomassie Brilliant Blue and imaged (ChemiDoc, Bio-Rad, Hercules, CA, USA).

### Tyrosine aminotransferase assay

Reactions were initiated by adding tyrosine substrate (0–8 mM) to the remaining components, for a final concentration of 100 mM Tris–HCl pH 8.5, 100 mM α-ketoglutarate, 0.2 mM PLP, 1 ng/µL enzyme, in a 300-µL final reaction volume. The reaction mixtures were incubated at 30°C for 5 min and terminated by adding sodium hydroxide (final concentration of 400 mM). After incubation at room temperature for 30 min in the dark, product formation was measured spectrophotometrically by absorption at 331 nm using the Infinite M Plex plate reader (Tecan, Männedorf, Switzerland). The reaction with no tyrosine added was used as the background control. Kinetic parameters were calculated with GraphPad. All enzymatic assays were performed under conditions where product formation increased proportionally to enzyme concentration and reaction time.

### Chl content and *Fv/Fm* analysis

Chl content of the T-DNA insertion lines was determined as described previously (Porra et al., 1989). *Fv/Fm*, which corresponds to the potential quantum yield of the photochemical reactions of PSII, was also measured as described previously (Oh et al., 1996).

### RT-qPCR analysis 

Total RNA was extracted from rosette leaves using TRIzol reagent (Invitrogen, Waltham, MA, USA). First-strand cDNA synthesis was performed as per the manufacturer’s instructions using the PrimeScript RT Reagent Kit with gDNA Eraser (Takara, Shiga, Japan). qPCR was performed on an ABI Prism^®^ 7900 HT real-time PCR system (Applied Biosystems/Life Technologies, Darmstadt, Germany) in 384-well PCR plates. The RT‐qPCR data were analyzed using the 2^−ΔΔCt^ analysis method according to Bustin et al. (2009).

### α-galactosidase, β-galactosidase, and β-glucosidase activity assay of BGLU42 in *E. coli*

The *E. coli* mutant strain BW25113 (*ΔmelA*) α-galactosidase activity was obtained from the KeiO collection; *LacZ* (the ß-galactosidase orthologous gene in *E. coli*) was already disrupted in this strain (Baba et al., 2006). α-galactosidase and β-glucosidase activities were determined by the production of *p*-nitrophenol (yellow color in solution) from the hydrolysis of *p*-nitrophenyl *α-*d-galactopyranosides (Sigma, St Louis, MO, USA; 7493-95-0) and of the hydrolysis of *p-*nitrophenyl *β-*d*-*glucopyranoside (without phosphorylation β-glucoside, Sigma, 2492-87-7). β-galactosidase activity was observed by the production of a dark blue precipitate from the hydrolysis of 5-bromo-4-chloro-3-indolyl-β-d-galactopyranoside on plates. For α-galactosidase activity, the cultures were incubated in reaction mixture containing 3 mM *p*-nitrophenyl *α-*d-galactopyranosides, 3 mM MnSO_4_, 0.15 mM NAD^+^, 5 mM dithiothreitol and 10 mM Tris–HCl, pH 8.1 in a shaking bath at 30°C. The reaction was stopped by the addition of Na_2_CO_3_ and EDTA to a final concentration of 0.25 M and 40 mM, respectively (Nagao et al., 1988). For β-glucosidase activity, the cultures were incubated with 50 mM phosphate citrate buffer (pH 6.0) containing 0.5 mg/mL *p-*nitrophenyl *β-*d*-*glucopyranoside in a shaking bath for 1 h. The reaction was stopped by the addition of an equal volume of 1 M Na_2_CO_3_ (Love and Streiff, 1987).

### Accession numbers

The raw metabolomics data (GC/LC data) in this article can be found in Zenodo (https://zenodo.org/) (doi: 10.5281/zenodo.5169901, 10.5281/zenodo.5176385, 10.5281/zenodo.5170287, 10.5281/zenodo.5170211, 10.5281/zenodo.5169932, and 10.5281/zenodo.5169912). Accession numbers based on The Arabidopsis Information Resource (https://www.arabidopsis.org) for all genes examined in this study are as follows: *TAT1* (At5g53970), *THA1* (At1g08630), *BGLU42* (At5g36890), *bHLH093* (At5g65640), *GTE7* (At5g65630), *IAA9* (At5g65670), *AVT1B* (At3g54830), *BCAT2* (At1g10070), *ALANINE:GLYOXYLATE AMINOTRANSFERASE 2* (*AGT2*; At4g39660), *TyrDC* (At4g28680), *P5CS1* (At2g39800), *LACS9* (At1g77590), *NAOD* (At4g17830), *ARGINASE 1* (At4g08900), *SHN2* (At5g11190), *CHY4* (At4g31810), *ProDH* (At3g30775), *AO/FDH* (At5g14760/At5g14780), *KCS4* (At1g19440), *PLDγ2* (At4g11830), *TAG1* (At2g19450), *KCS13* (At2g46720), *ACX3/6* (At1g06290/At1g06310), *LYSOPHOSPHATIDYLCHOLINE ACYLTRANSFERASE 1* (*LPCAT1*; At1g12640), *DELTA9 DESATURASE 4.2 (ADS4.2*; At1g06360), *LYSOPHOSPHATIDYLETHANOLAMINE ACYLTRANSFERASE 1* (*LPEAT1*; At1g80950), and *ACTIN2* (At3g18780).

## Supplemental data 

The following materials are available in the online version of this article.

**Supplemental Figure S1.** Manhattan plots of genome-wide associations for Fv*/Fm* values and Chl content in different datasets.

**Supplemental Figure S2.** Phenotype analysis of *bcat2-1* and *bcat2-2* mutants.

**Supplemental Figure S3.** Metabolite networks for different datasets.

**Supplemental Figure S4.** LD plot for the locus associated with proline and DAG 34:2 for the 0 days dataset and leucine for the 3 days dataset.

**Supplemental Figure S5.** Manhattan and LD plots of galactinol contents and enzyme activity analysis of BGLU42.

**Supplemental Figure S6.** Manhattan and LD plots of trehalose contents and *TRE1* expression between different genotypes.

**Supplemental Figure S7.** Manhattan and LD plots of glycine contents and gene expression or *AVT1B* and TMHMM analysis of AVT1B.

**Supplemental Figure S8.** Manhattan and LD plots of ornithine contents and ornithine content analysis of *bHLH093* and *IAA9* T-DNA insertion mutants.

**Supplemental Figure S9.** Manhattan and LD plots of tyrosine, *Fv/Fm* result of *tat1-1* and *tat1-2* mutants and protein stability and purity of the two types of TAT1 proteins.

**Supplemental Figure S10.** Manhattan and LD plots of threonine contents, validation and leaf phenotype result of *tha1-2* and *tha1-3* mutants and the expression of *THA1* into darkness treatment.

**Supplemental Figure S11.** Summary of Chl content and *Fv/Fm* of *TAT1*, *THA1*, *Bglu42*, and *BCAT2* mutants under darkness condition.

**Supplemental Data Set S1.** Normalized data for GWAS.

**Supplemental Data Set S2.** GWAS based on imputed 1.2M SNPs of individual results.

**Supplemental Data Set S3.** GWAS based on multivariate models.

**Supplemental Data Set S4.** GWAS based on 200K SNPs Affymetrix Chip data.

**Supplemental Data Set S5.** CV (coefficient of variation) and *H^2^* of metabolites.

**Supplemental Data Set S6.** Cluster information of metabolic shift patterns for the three time points.

**Supplemental Data Set S7.** Overlapping loci information of darkness-related GWAS results.

**Supplemental Data Set S8.** List of Arabidopsis accessions and related information.

**Supplemental Data Set S9.** Summary of statistical tests.

**Supplemental Table S1.** LD analysis of SNPs in *AVT1B* gene region.

**Supplemental Table S2.** LD analysis of SNPs in the *TAT1* gene region.

**Supplemental Table S3.** LD analysis of SNPs in the *THA1* gene region.

**Supplemental Table S4.** T-DNA lines used in this study.

**Supplemental Table S5.** Primers used in this study.

## Supplementary Material

koab251_Supplementary_Data

## Notes

F.Z., Y.C., and A.R.F. designed the research. F.Z., S.A., and Y.B. performed and analyzed the metabolome-related analysis. K.K., H.M., and F.Z. performed the TAT1 enzymatic activity assay. F.Z., S.A., H.T., Z.N., T.N., H.L., W.W., and J.Y. performed the mGWAS analysis. F.Z. and A.R.F. wrote the manuscript with input from all authors.

The author responsible for distribution of materials integral to the findings in this article in accordance with the policy described in the Instructions for Authors (https://academic.oup.com/plcell) is Alisdair R. Fernie (fernie@mpimp-golm.mpg.de).
