# Supplementary material for: Genome-wide association of the metabolic shifts underpinning dark-induced senescence in Arabidopsis
Source: Plant Cell. 2021 Oct 8;34(1):557–78. doi: 10.1093/plcell/koab251 (PMC8774053; doi:10.1093/plcell/koab251)
Supplement: koab251_Supplementary_Data [file koab251_supplementary_data.zip › Supplemental Figure S111 and Table S15.pdf]

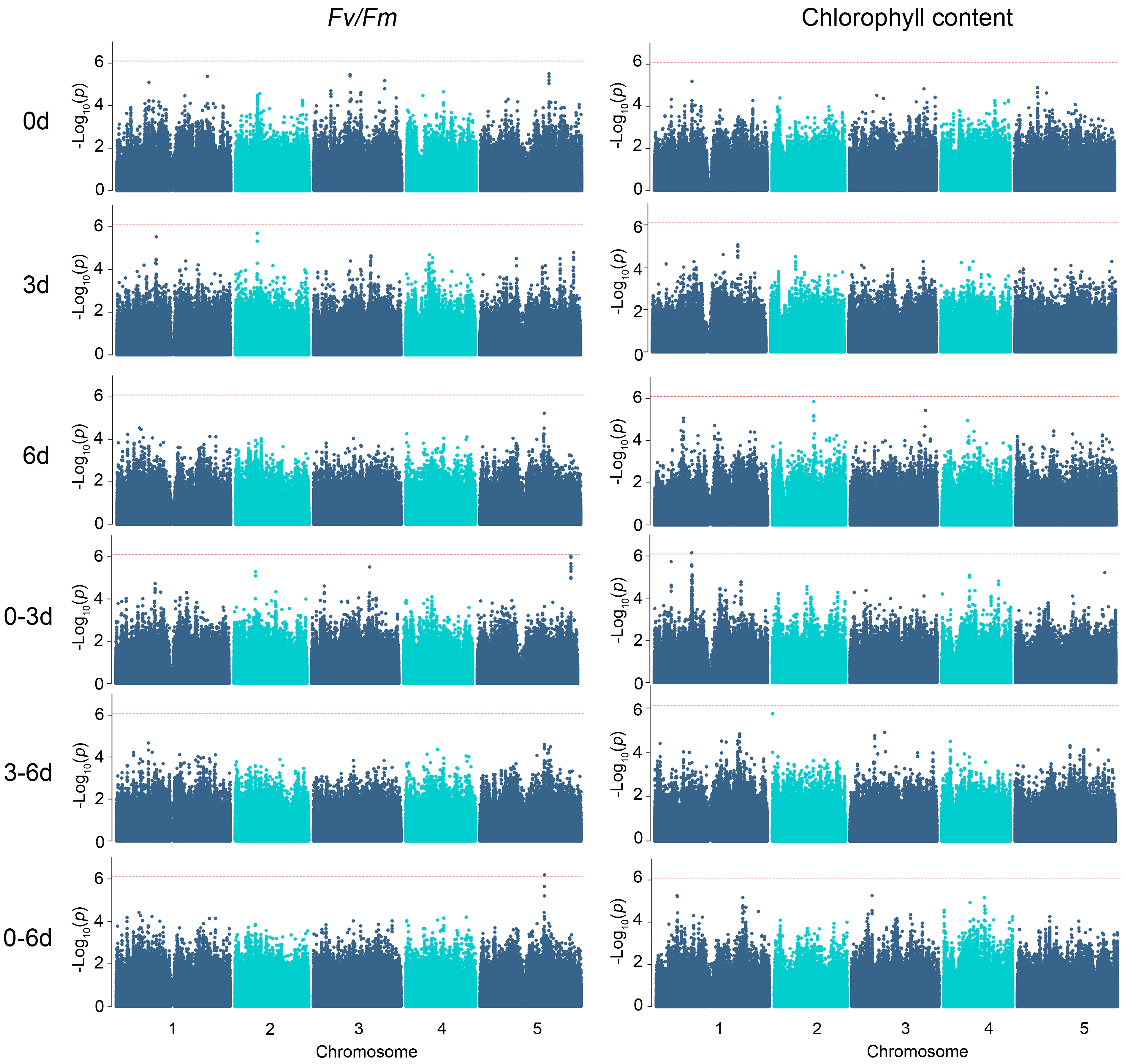

Supplemental Figure S1.The Manhattan plots of genome-wide associations of for *Fv/Fm* values and chlorophyll content in different datasets. Supports Figure 2.

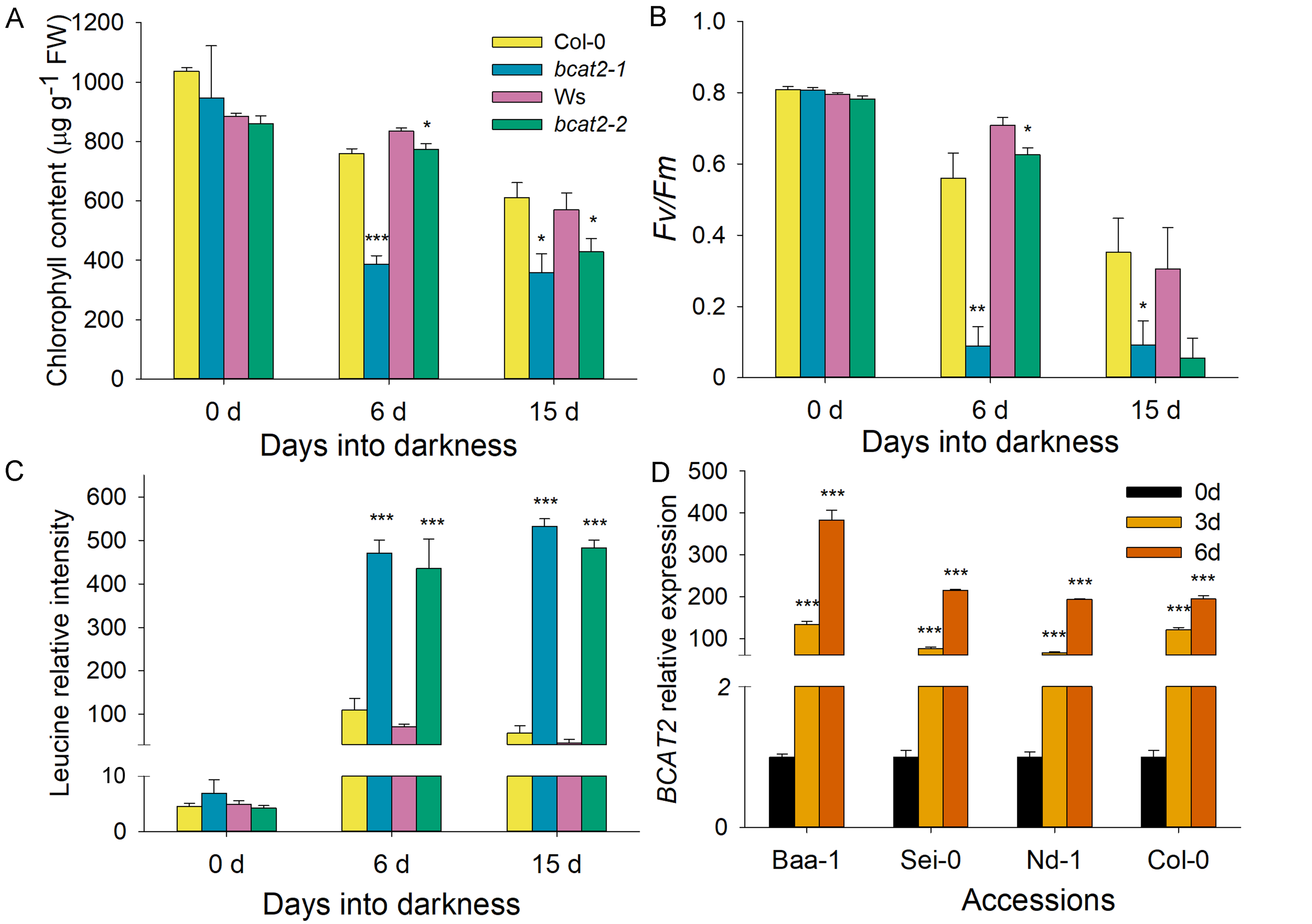

Supplemental Figure S2. The phenotype analysis of *bcat2-1* and *bcat2-2* mutants. Supports Figure 2.
(A) Chlorophyll content of *bcat2-1* and *bcat2-2* mutants under darkness. Data are shown as means ± SD (n= three biological replicates. Asterisks indicate statistically significant differences relative to Col-0 or Ws, as determined by two-tailed Student's *t*-test: *, $p < 0.05$; ***, $p < 0.001$.
(B) Fv/Fm values of *bcat2-1* and *bcat2-2* mutants under darkness. Data are shown as means ± SD (n= three biological replicates. Asterisks indicate statistically significant differences relative to Col-0 or Ws, as determined by two-tailed Student's *t*-test: *, $p < 0.05$; **, $p < 0.01$.
(C) Leucine content of *bcat2-1* and *bcat2-2* mutants upon extended darkness for 6 d to 15 d. Data are shown as means ± SD (n= six biological replicates. Asterisks indicate statistically significant differences relative to Col-0 or Ws, as determined by two-tailed Student's *t*-test: ***, $p < 0.001$.
(D) Relative BCAT2 expression levels upon darkness exposure of different accessions. Data are shown as means ± SD (n= three biological replicates. Asterisks indicate statistically significant differences relative to 0 d, as determined by two-tailed Student's *t*-test: ***, $p < 0.001$.

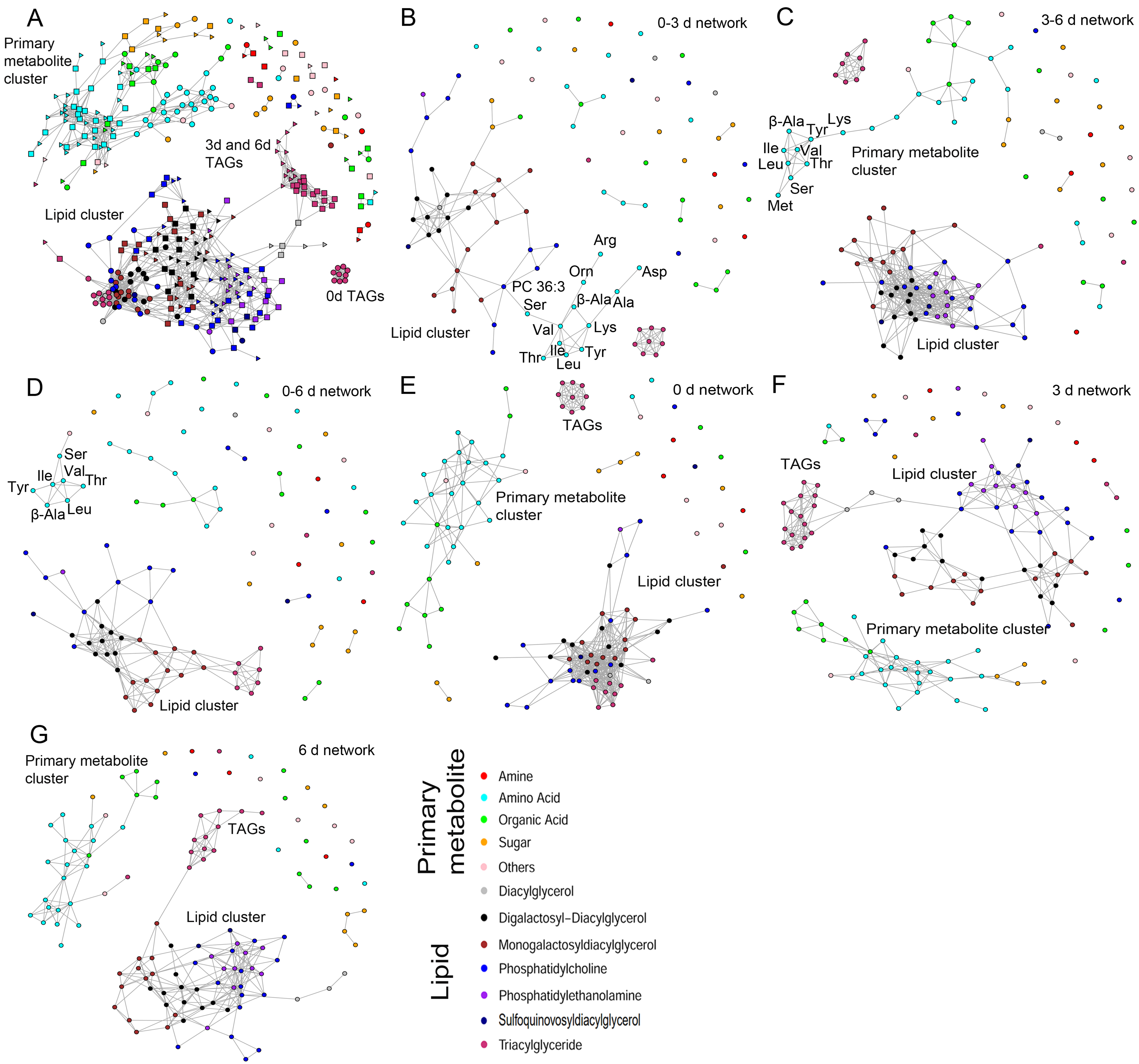

Supplemental Figure S3.The metabolite network different datasets. Supports Figure 2. Metabolite networks of 0/3/6 d (A), 0-3 d (B), 3-6 d (C), 0-6 d (D), 0 d (E), 3 d (F) and 6 d (G) datasets. Nodes that correlate with each other are linked by gray edges. Edges between nodes represent correlations identified as significant at $r > 0.5$ and p_{adj} value <0.05.

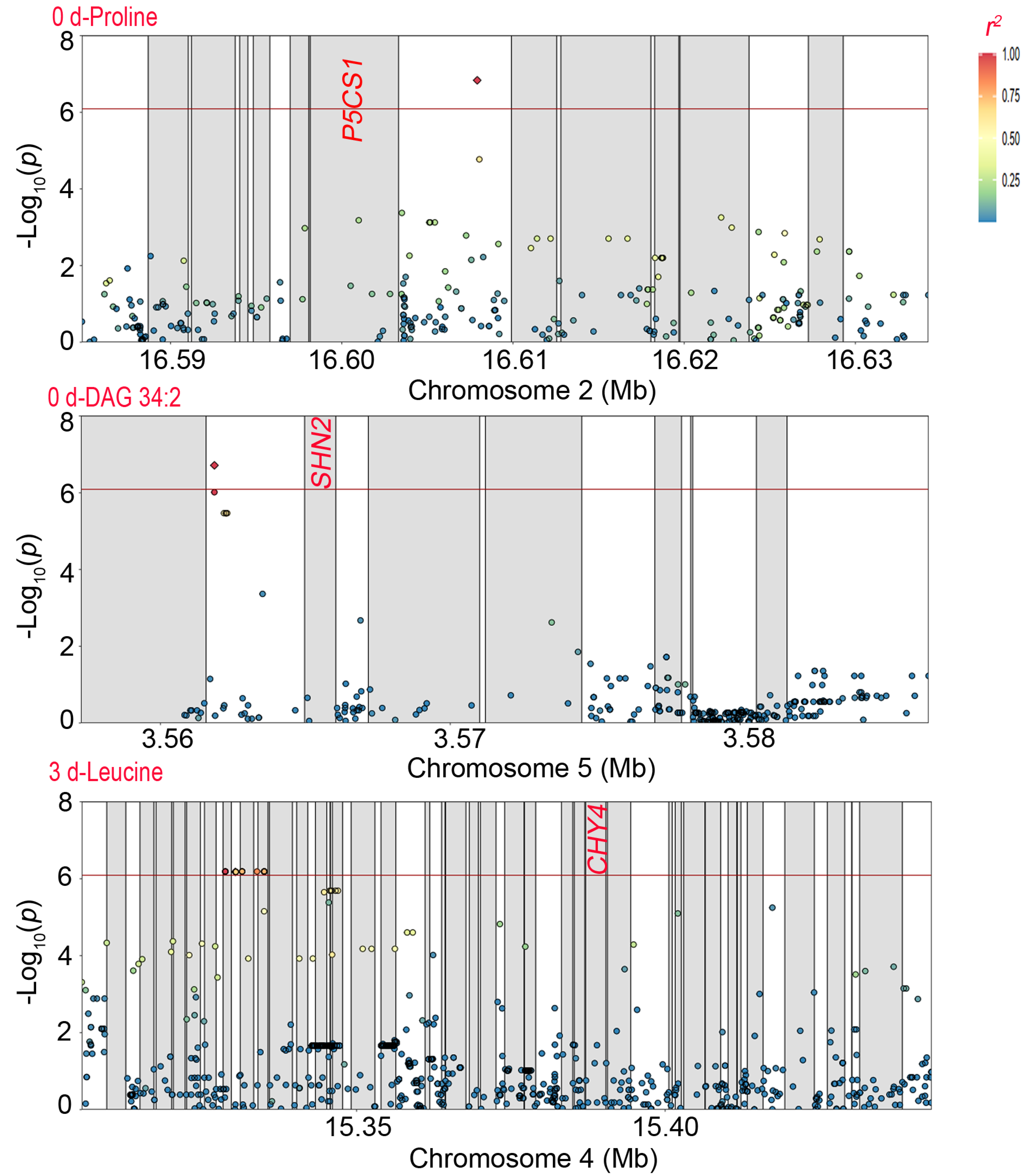

Supplemental Figure S4. The linkage disequilibrium (LD) plot for the locus associated with proline and DAG 34:2 for the 0 d dataset and leucine for the 3 d dataset. Supports Figure 2.
Linkage disequilibrium (LD) plot based on imputed 1.2 M SNP data for the associations with proline and DAG 34:2 contents for the 0 d dataset and leucine contents for the 3 d dataset. The *x*- and *y*-axes are as in Figure 3A. Each gray block denotes a gene and each circle indicates a SNP, with the lead SNP (with lowest *p*-value) shown as a red diamond. The color of each circle reflects the r^2 value with the lead SNP.

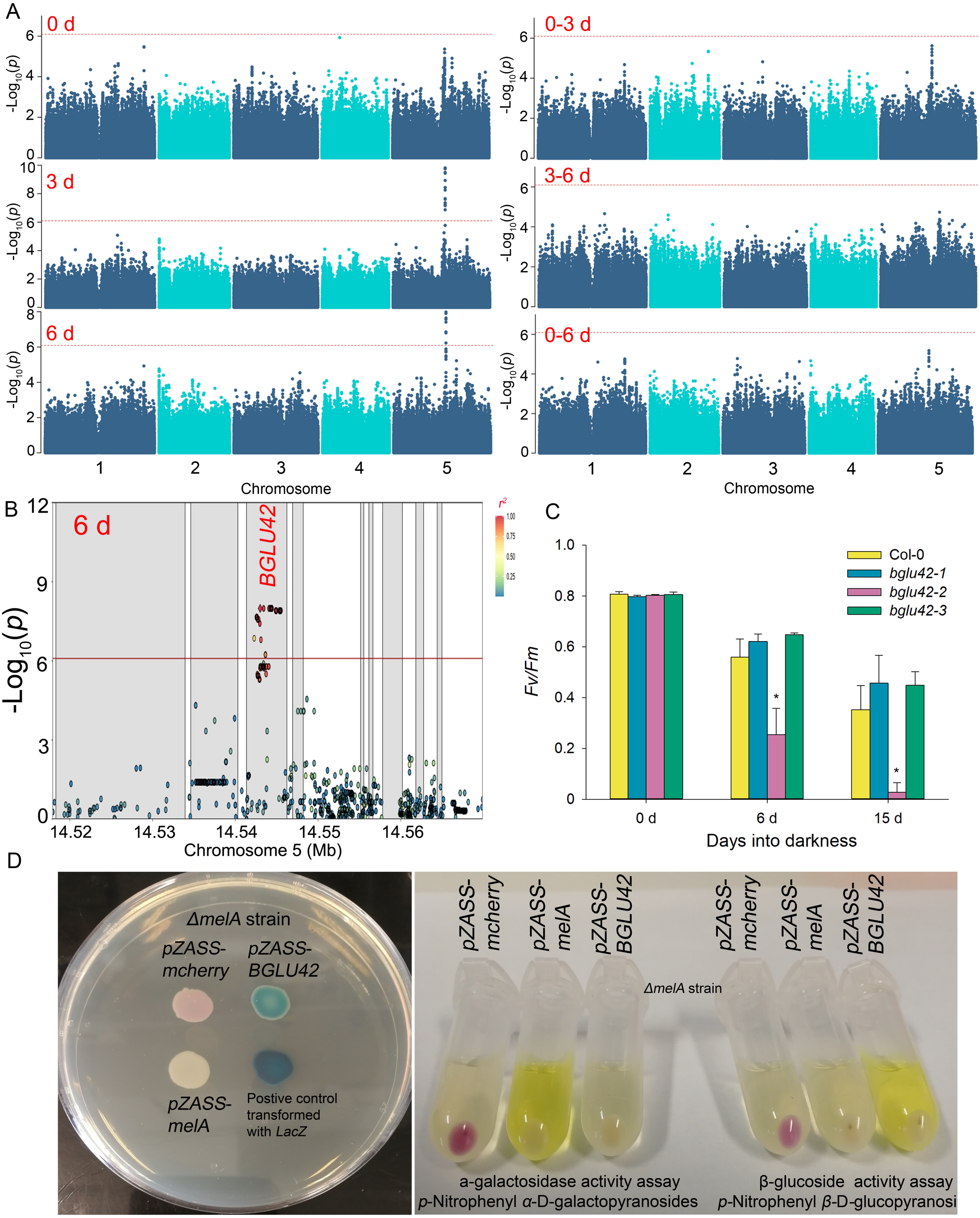

Supplemental Figure S5. The Manhattan and linkage disequilibrium (LD) plots of galactinol and enzyme activity analysis of BGLU42. Supports Figure 3.
(A) Manhattan plots of galactinol contents for the different datasets.
(B) Linkage disequilibrium (LD) plot based on the imputed 1.2 M SNP data for the associations with galactinol contents for the 6d dataset.
(C) *Fv/Fm* values of *bglu42-1*, *bglu42-2* and *bglu42-3* mutants in extended darkness. Data are shown as means ± SD (n= three biological replicates. Asterisks indicate statistically significant differences relative to Col-0, as determined by Student's two-tailed *t*-test: *, $p < 0.05$.
(D) β-galactosidase (left, blue color), α-galactosidase (middle, yellow color) and β-glucosidase (right, yellow color) enzyme activity in the loss-function mutant (*ΔmelA*) *E. coli* strain transformed with *pZASS-mcherry*, *pZASS-melA* and *pZASS-BGLU42*.

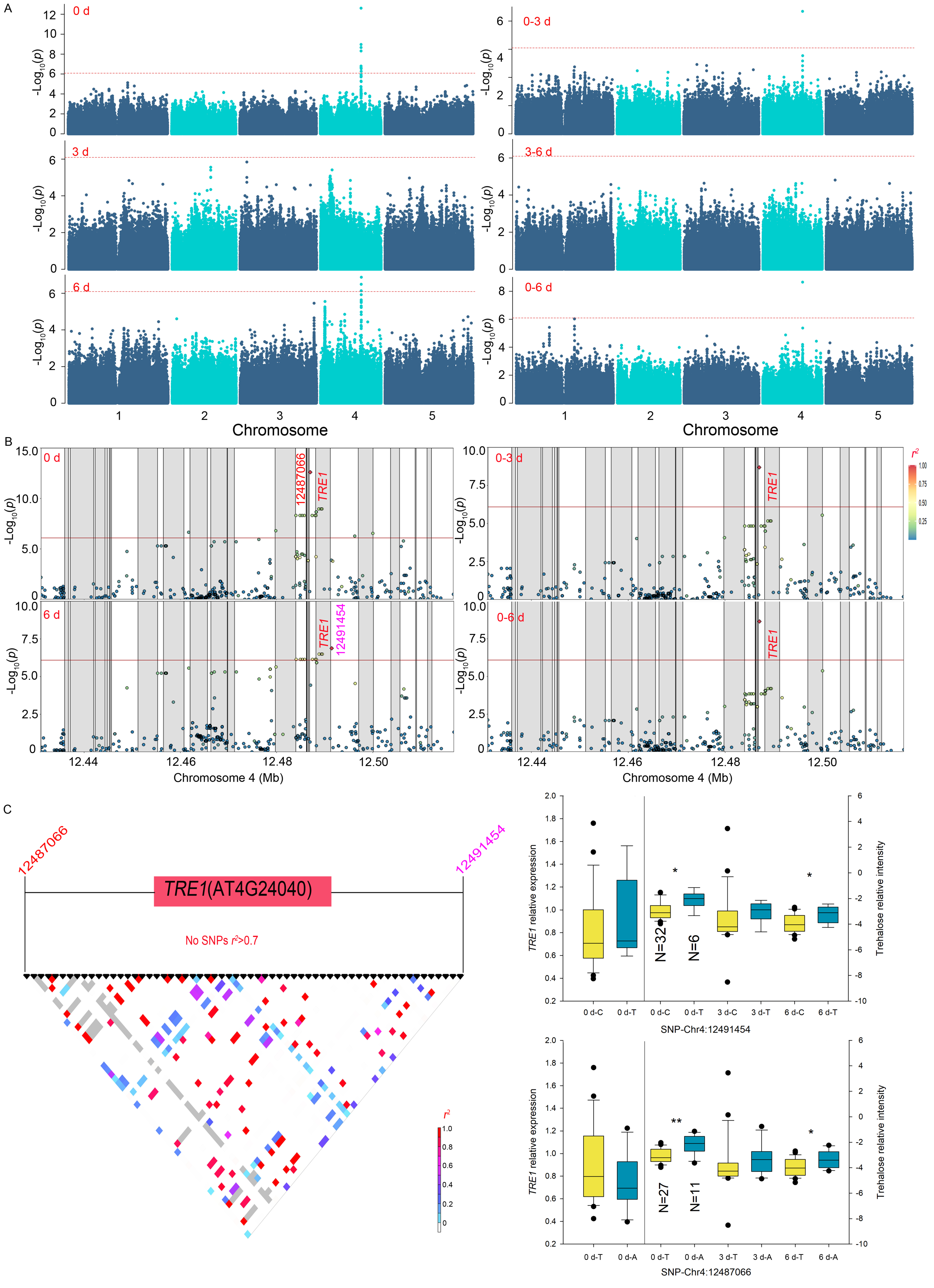

Supplemental Figure S6. Manhattan and linkage disequilibrium (LD) plots of trehalose and *TRE1* expression between different genotypes. Supports Figure 2.
(A) Manhattan plots of trehalose contents for the different datasets.
(B) Linkage disequilibrium (LD) plot based on the imputed 1.2 M SNP data for the association with trehalose levels for the 0 d, 6 d, 0-3 d and 0-6 d datasets.
(C) LD plot based on the SNPs between the lead SNPs (Chr4:12487066 and Chr4:12491454) and *TRE1* nearby genes region in the 1001 Arabidopsis genomes database. The number of accessions with each genotype is given for the 0 d phenotypic values. The boxplots represent the interquartile range, the solid horizontal line represents the median, the whiskers represent 1.5 x interquartile range, and the black circles represent outliers. Asterisks indicate statistical significance, as determined by two-tailed Student's *t*-test: *, $p < 0.05$; **, $p < 0.01$.

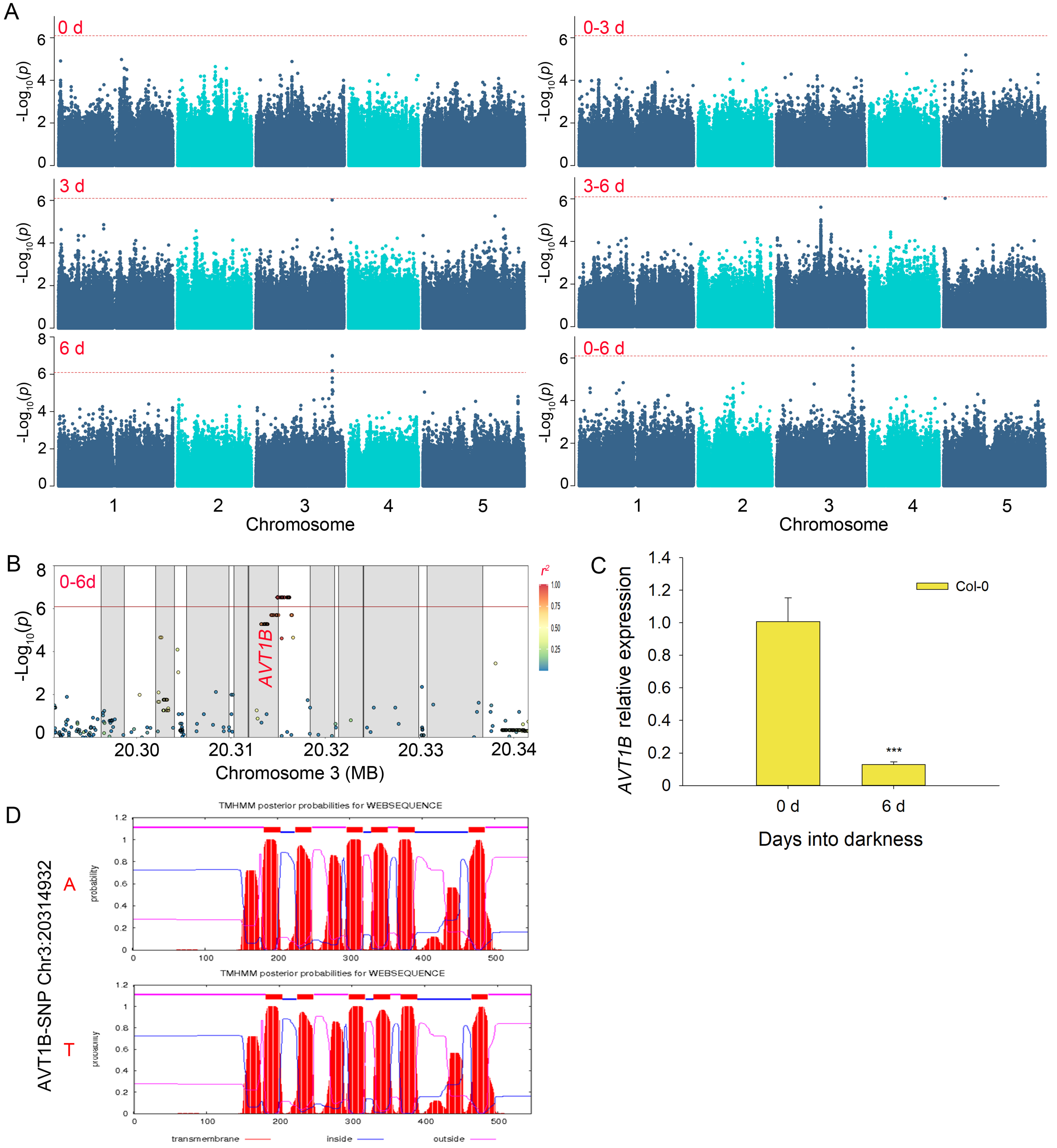

Supplemental Figure S7. Manhattan and linkage disequilibrium (LD) plots of glycine and gene expression and TMHMM analysis of AVT1B. Supports Figure 3.

(A) Manhattan plots of glycine contents for the different datasets.

(B) Linkage disequilibrium (LD) plot based on imputed 1.2 M SNP data for the locus associated with glycine contents for the 0-6 d datasets.

(C) Relative *AVT1B* expression levels upon extended darkness in Col-0. Data are shown as means ± SD (n= three biological replicates. Asterisks indicate statistically significant differences relative to 0 d, as determined by two-tailed Student's *t*-test: **, $p < 0.01$.

(D) TM-HMM results of AVT1B-SNP Chr3:20314932-A and AVT1B-SNP Chr3:20314932-T.

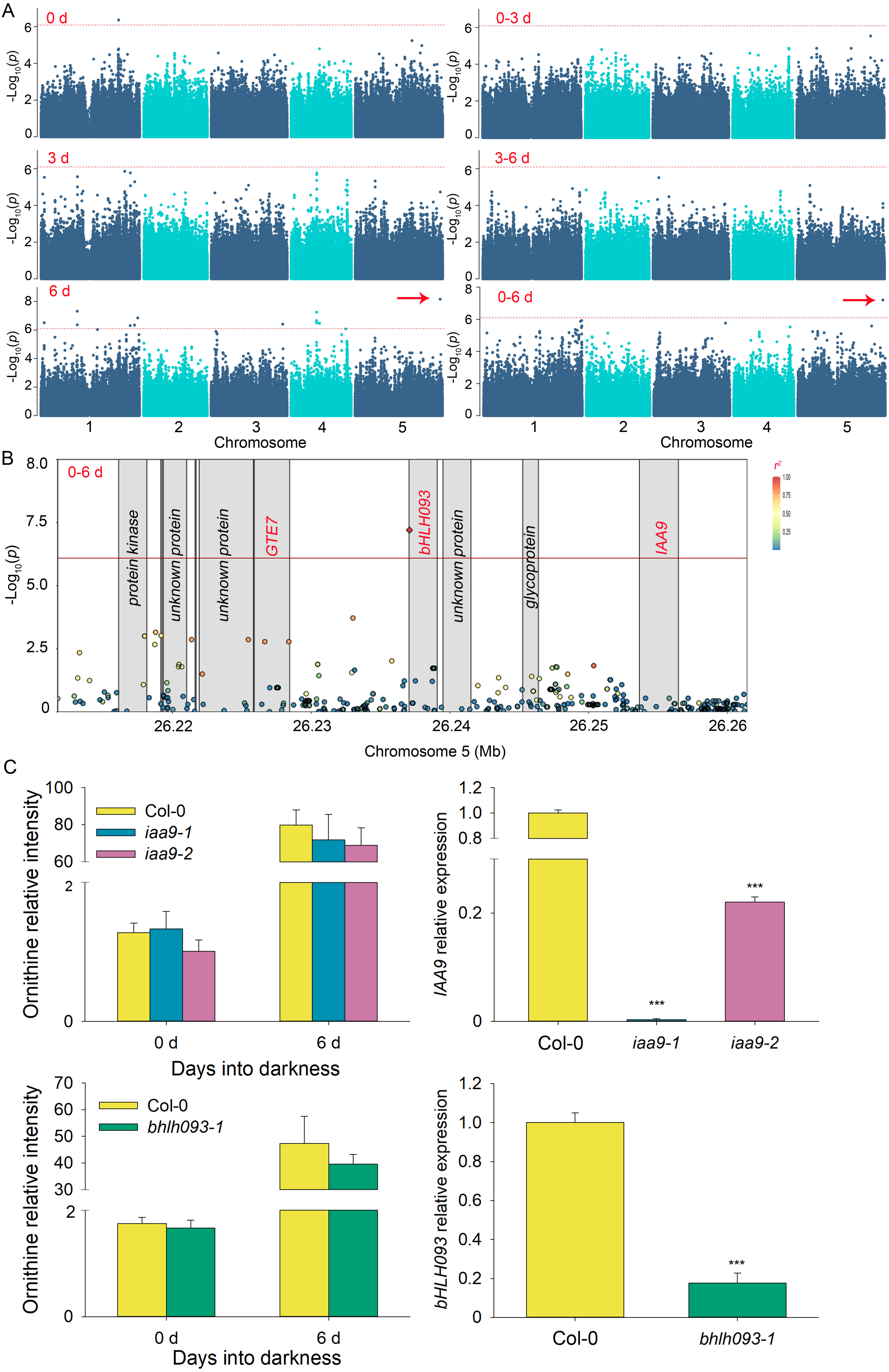

Supplemental Figure S8. Manhattan and linkage disequilibrium (LD) plots of ornithine and ornithine content analysis of *bHLH093* and *IAA9* T-DNA insertion mutants. Supports Figure 4.

(A) Manhattan plots of ornithine contents for the different datasets.

(B) Linkage disequilibrium (LD) plot based on the imputed 1.2 M SNP data for the association with ornithine contents for the 0-6 d dataset.

(C) Ornithine content and relative expression levels of bHLH093 and IAA9 in T-DNA insertion mutants. Data are shown as means ± SD (n= five to six biological replicates. Asterisks indicate statistically significant differences relative to Col-0, as determined by two-tailed Student's *t*-test: ***, $p < 0.001$.

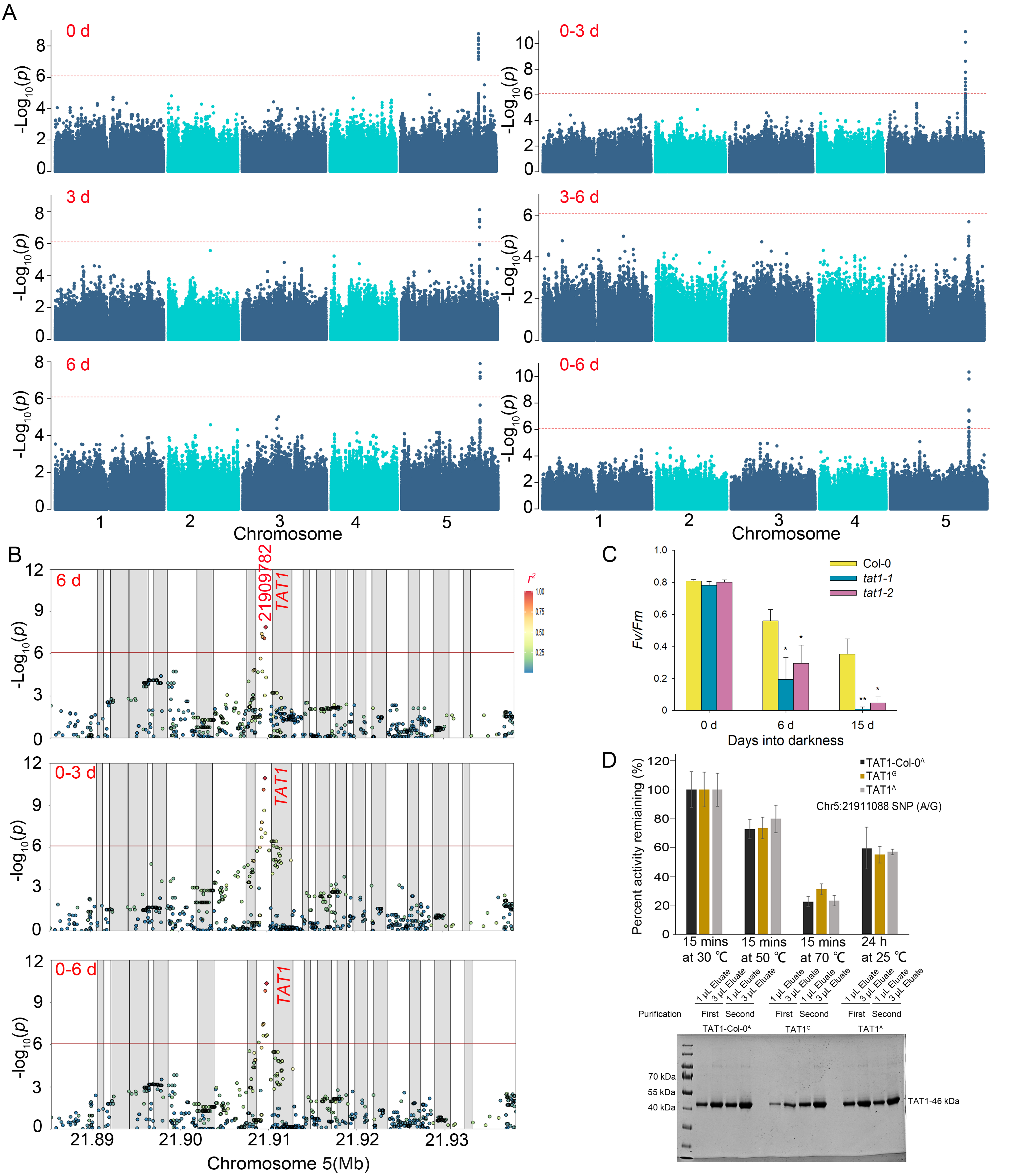

Supplemental Figure S9. Manhattan and linkage disequilibrium (LD) plots of tyrosine, *Fv/Fm* result of *tat1-1* and *tat1-2* mutants and protein stability and purity of the two types of TAT1 proteins. Supports Figure 5.

(A) Manhattan plots of tyrosine contents for the different datasets.

(B) Linkage disequilibrium (LD) plot based on the imputed 1.2 M SNP data for the association with tyrosine contents at 6 d, 0-3 d and 0-6 d datasets.

(C) *Fv/Fm* values of *tat1-1* and *tat1-2* mutants during extended darkness. Data are shown as means ± SD (n= three biological replicates. Asterisks indicate statistically significant differences relative to Col-0, as determined by two-tailed Student's *t*-test: *, $p < 0.05$; **, $p < 0.01$.

(D) Stability (up) and protein purity (down) of the two types of TAT1 proteins based on SNP-Chr5:21911088. Data are shown as means ± SD (n= four biological replicates). Polyhistidine tagged TAT1-Col-0^{A}, TAT1^{G} and TAT1^{A} were purified using imidazole metal affinity chromatography and desalted. For each enzyme, two independent purifications were prepared. 1 and 3 μL of desalted eluate were resolved using SDS-PAGE.

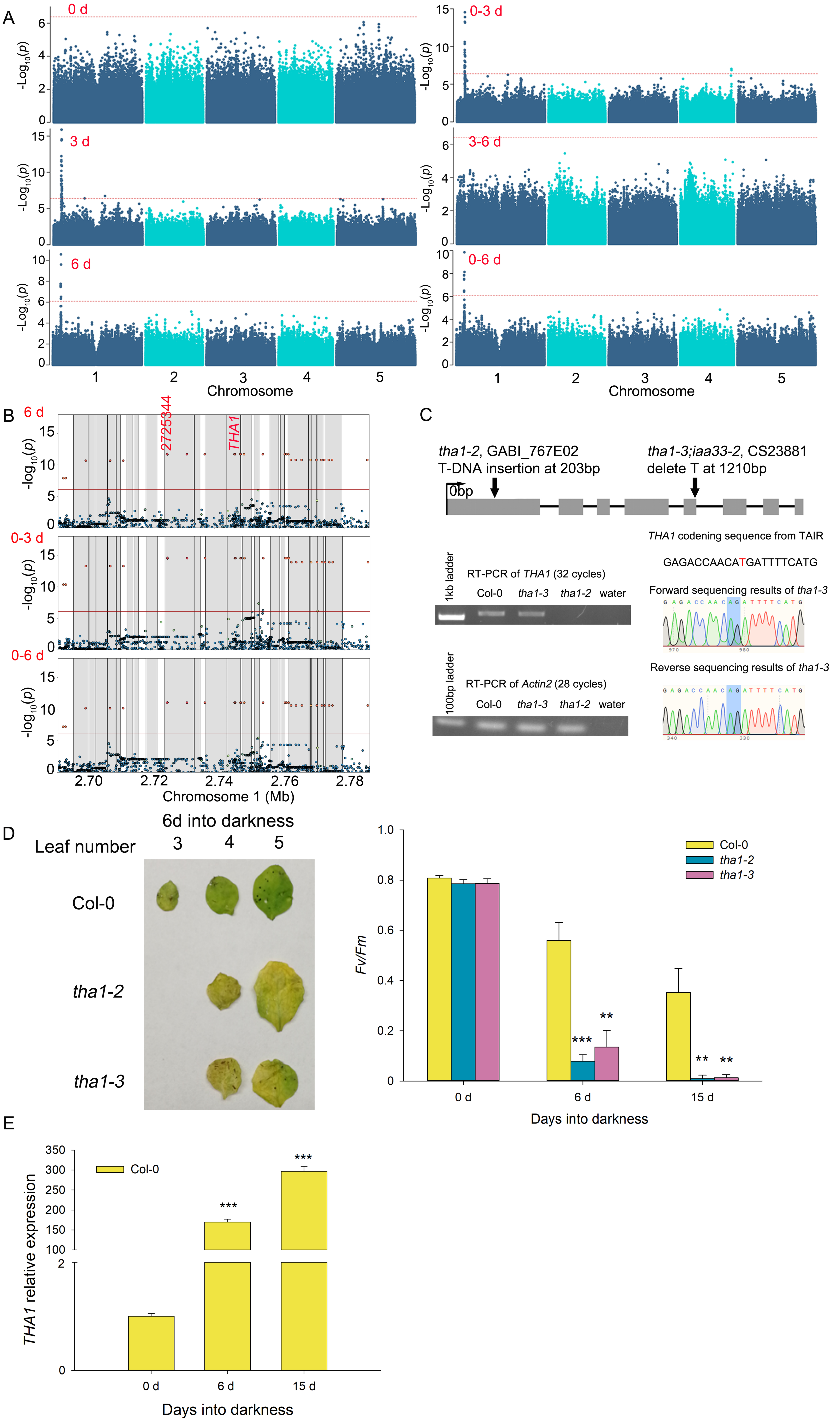

Supplemental Figure S10. Manhattan and linkage disequilibrium (LD) plots of threonine, validation and leaf phenotype result of *tha1-2* and *tha1-3* mutants and the expression of *THA1* after darkness treatment. Supports Figure 6.

(A) Manhattan plots of threonine contents for the different datasets.

(B) Linkage disequilibrium (LD) plot based on the imputed 1.2 M SNP data for the association with threonine contents for the 6 d, 0-3 d and 0-6 d datasets.

(C) RT-PCR analysis of *tha1-2* and *tha1-3* mutants and the one base deletion sequence results of the *tha1-3* mutant.

(D) Leaf phenotype and Fv/Fm values of tha1-2 and tha1-3 mutants upon extended darkness. Data are shown as means ± SD (n= three biological replicates). Asterisks indicate statistically significant differences relative to Col-0, as determined by two-tailed Student's *t*-test: **, $p < 0.01$; ***, $p < 0.001$.

(E) Relative THA1 expression levels under darkness treatment in Col-0. Data are shown as means ± SD (n= three biological replicates). Asterisks indicate statistically significant differences relative to Col-0, as determined by two-tailed Student's *t*-test: ***, $p < 0.001$.

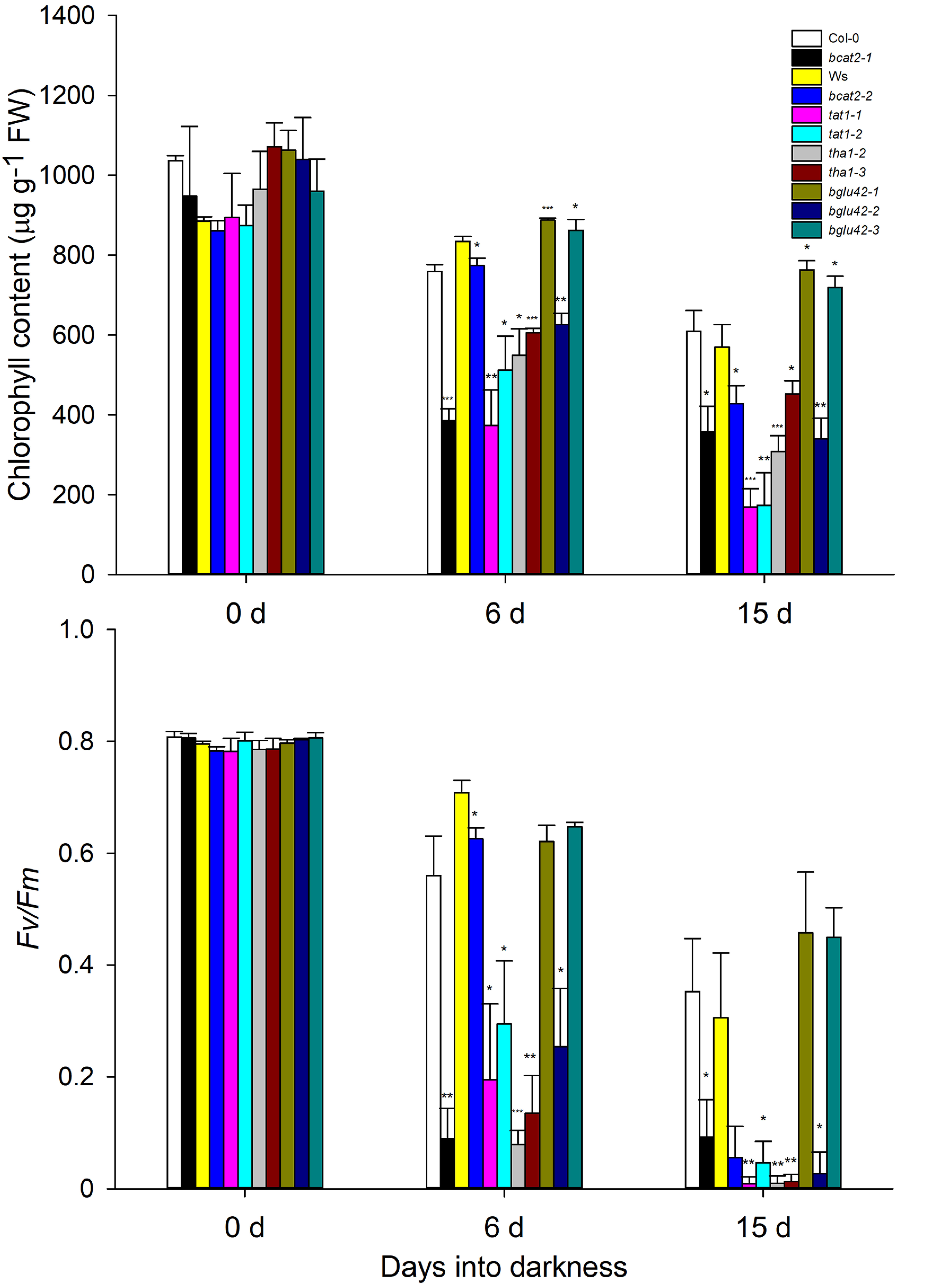

Supplemental Figure S11. Summary of chlorophyll contents and *Fv/Fm* values for *tat1*, *tha1*, *bglu42* and *bcat2* mutants under darkness. Supports Figure 3, 5 and 6.
Results are from Figure 3B, Figure 5B, Figure 6B, Supplemental Figure S2, S9C and S10D. Data are shown as means ± SD (n= three biological replicates). Asterisks indicate statistically significant differences relative to Col-0, as determined by two-tailed Student's *t*-test: *, $p < 0.05$; **, $p < 0.01$; ***, $p < 0.001$.

Supplemental Tables.

Supplemental Table S1. LD analysis of SNPs in the *AVT1B* gene region.

**Chromosome**	**Position**	**SNP**	**Chromosome**	**Position**	**SNP**	r^2	**effect type**	**Amino Acid Change**
3	20,314,932	A:T	3	20,313,208	G:A	0.81	intron variant	
3	20,314,932	A:T	3	20,313,227	A:T	0.75	intron variant	
3	20,314,932	A:T	3	20,313,250	A:G	0.78	intron variant	
3	20,314,932	A:T	3	20,313,253	A:T	0.79	intron variant	
3	20,314,932	A:T	3	20,313,255	G:A	0.80	intron variant	
3	20,314,932	A:T	3	20,313,348	C:G	0.81	synonymous variant	
3	20,314,932	A:T	3	20,313,438	T:A	0.82	synonymous variant	
3	20,314,932	A:T	3	20,313,588	A:G	0.74	intron variant	
3	20,314,932	A:T	3	20,313,618	A:T	0.73	intron variant	
3	20,314,932	A:T	3	20,313,670	G:C	0.78	intron variant	
3	20,314,932	A:T	3	20,313,767	C:A	0.76	intron variant	
3	20,314,932	A:T	3	20,313,771	A:T	0.75	intron variant	
3	20,314,932	A:T	3	20,313,773	C:A	0.75	intron variant	
3	20,314,932	A:T	3	20,313,775	A:G	0.73	intron variant	
3	20,314,932	A:T	3	20,313,786	C:A	0.75	intron variant	
3	20,314,932	A:T	3	20,313,788	A:C	0.77	intron variant	
3	20,314,932	A:T	3	20,313,795	T:C	0.77	intron variant	
3	20,314,932	A:T	3	20,313,815	T:A	0.77	intron variant	
3	20,314,932	A:T	3	20,313,826	A:G	0.79	synonymous variant	
3	20,314,932	A:T	3	20,313,892	A:C	0.82	intron variant	
3	20,314,932	A:T	3	20,313,899	C:A	0.81	intron variant	
3	20,314,932	A:T	3	20,313,962	G:A	0.75	synonymous variant	
3	20,314,932	A:T	3	20,314,268	A:T	0.86	intron variant	
3	20,314,932	A:T	3	20,314,364	A:T	0.86	intron variant	
3	20,314,932	A:T	3	20,314,427	C:T	0.87	intron variant	
3	20,314,932	A:T	3	20,314,570	T:A	0.86	missense variant	Thr-126-Ser
3	20,314,932	A:T	3	20,314,807	T:C	0.86	intron variant	
3	20,314,932	A:T	3	20,314,826	T:A	0.86	intron variant	
3	20,314,932	A:T	3	20,314,854	A:T	0.86	synonymous variant	
3	20,314,932	A:T	3	20,314,889	G:A	0.86	synonymous variant	
3	20,314,932	A:T	3	20,314,941	G:T	1	missense variant	Asp-30-Glu
3	20,314,932	A:T	3	20,315,034	C:T	0.84	intergenic region	
3	20,314,932	A:T	3	20,315,048	A:T	0.97	intergenic region	
3	20,314,932	A:T	3	20,315,213	C:T	0.96	intergenic region	

Chromosome	Position	SNP	Chromosome	Position	SNP	r^2	effect type	Amino Acid Change
3	20,314,932	A:T	3	20,315,364	T:C	0.95	intergenic region	
3	20,314,932	A:T	3	20,315,376	T:G	0.95	intergenic region	
3	20,314,932	A:T	3	20,315,387	T:C	0.95	intergenic region	
3	20,314,932	A:T	3	20,315,391	C:T	0.97	intergenic region	
3	20,314,932	A:T	3	20,315,414	G:A	0.95	intergenic region	
3	20,314,932	A:T	3	20,315,429	C:A	0.94	intergenic region	
3	20,314,932	A:T	3	20,315,459	C:A	0.92	intergenic region	
3	20,314,932	A:T	3	20,315,461	T:G	0.91	intergenic region	
3	20,314,932	A:T	3	20,315,476	G:T	1	intergenic region	
3	20,314,932	A:T	3	20,315,477	A:C	1	intergenic region	
3	20,314,932	A:T	3	20,315,648	G:A	0.92	intergenic region	
3	20,314,932	A:T	3	20,315,668	T:G	0.96	intergenic region	
3	20,314,932	A:T	3	20,315,926	C:T	0.87	intergenic region	
3	20,314,932	A:T	3	20,316,023	C:T	0.89	intergenic region	
3	20,314,932	A:T	3	20,316,036	T:A	0.92	intergenic region	
3	20,314,932	A:T	3	20,316,074	A:C	0.96	intergenic region	
3	20,314,932	A:T	3	20,316,076	A:G	1	intergenic region	
3	20,314,932	A:T	3	20,316,085	G:T	0.96	intergenic region	
3	20,314,932	A:T	3	20,316,088	T:G	0.95	intergenic region	
3	20,314,932	A:T	3	20,316,093	A:T	0.95	intergenic region	
3	20,314,932	A:T	3	20,316,100	T:A	0.95	intergenic region	
3	20,314,932	A:T	3	20,316,144	A:G	0.95	intergenic region	
3	20,314,932	A:T	3	20,316,177	A:T	0.95	intergenic region	
3	20,314,932	A:T	3	20,316,180	G:T	0.95	intergenic region	
3	20,314,932	A:T	3	20,316,195	C:T	0.94	intergenic region	
3	20,314,932	A:T	3	20,316,198	A:C	0.94	intergenic region	

Supplemental Table S2. LD analysis of SNPs in the *TAT1* gene region.

Chromosome	Position	SNP	Chromosome	Position	SNP	r^2	effect type	Amino Acid Change
5	21,909,782	A:G	5	21,909,421	C:T	0.75	intergenic region	
5	21,909,782	A:G	5	21,909,428	T:G	0.70	intergenic region	
5	21,909,782	A:G	5	21,909,631	T:C	0.87	intergenic region	
5	21,909,782	A:G		21,909,718	G:A	1	intergenic region	
5	21,911,088	A:G	5	21,910,251	A:G	0.81	intergenic region	
5	21,911,088	A:G	5	21,910,258	C:T	0.82	intergenic region	
5	21,911,088	A:G	5	21,910,260	T:G	0.82	intergenic region	
5	21,911,088	A:G	5	21,910,332	A:T	0.88	intergenic region	
5	21,911,088	A:G	5	21,910,469	C:T	0.71	intergenic region	
5	21,911,088	A:G	5	21,910,749	C:G	0.99	missense variant	Thr-25-Arg
5	21,911,088	A:G	5	21,910,762	T:A	0.97	missense variant	Asp-29-Glu
5	21,911,088	A:G	5	21,910,810	C:A	0.78	synonymous variant	
5	21,911,088	A:G	5	21,910,940	C:T	0.78	intron variant	
5	21,911,088	A:G	5	21,910,965	T:C	0.935	intron variant	
5	21,911,088	A:G	5	21,910,992	A:G	0.78	intron variant	
5	21,911,088	A:G	5	21,911,067	A:G	0.78	intron variant	
5	21,911,088	A:G	5	21,911,079	T:A	0.985	intron variant	
5	21,911,088	A:G	5	21,911,080	T:A	0.915	intron variant	
5	21,911,088	A:G	5	21,911,181	C:T	0.74	missense variant	Leu-96-Phe
5	21,911,088	A:G	5	21,911,183	T:G	0.73	synonymous variant	
5	21,911,088	A:G	5	21,911,188	A:T	0.73	missense variant	Gln-98-Leu
5	21,911,088	A:G	5	21,911,189	G:A	0.99	synonymous variant	
5	21,911,088	A:G	5	21,911,330	A:G	0.73	synonymous variant	
5	21,911,088	A:G	5	21,911,354	G:A	0.77	synonymous variant	
5	21,911,088	A:G	5	21,911,360	T:C	0.88	intron variant	
5	21,911,088	A:G	5	21,911,411	G:T	0.73	synonymous variant	
5	21,911,088	A:G	5	21,911,462	C:T	0.73	intron variant	
5	21,911,088	A:G	5	21,911,468	G:C	0.74	missense variant	Gln-191-His
5	21,911,088	A:G	5	21,911,520	T:G	0.74	intron variant	
5	21,911,088	A:G	5	21,911,720	A:T	0.935	synonymous variant	
5	21,911,088	A:G	5	21,911,744	C:T	0.74	synonymous variant	
5	21,911,088	A:G	5	21,911,888	A:C	0.75	synonymous variant	
5	21,911,088	A:G	5	21,912,746	A:T	0.82	downstream gene variant	

Supplemental Table S3. LD analysis of SNPs in the *THA1* gene region.

Chromosome	Position	SNP	Chromosome	Position	SNP	r^2	effect type	Amino Acid Change
1	2,725,344	A:G	1	2,746,095	A:G	0.92	intergenic region	
1	2,725,344	A:G	1	2,746,290	T:A	0.93	intergenic region	
1	2,725,344	A:G	1	2,747,871	G:T	0.83	intergenic region	
1	2,725,344	A:G	1	2,748,028	G:A	0.923	intergenic region	

Supplemental Table S4. T-DNA lines used in the present study.

AGI code	Allele name	Stock number
At5g53970	*tat1-1*	SALK_045398
	tat1-2	SALK_141402
At1g08630	*tha1-2*	GK-767E02
	tha1-3	CS23881
At5g36890	*bglu42-1*	SAIL_716_G04
	bglu42-2	SALK_034026
	bglu42-3	SALK_047096
At5g65640	*bhlh93-1*	SALK_121082
At5g65630	*GTE7*-RNAi-1	N23976
	GTE7-RNAi-2	N23977
At5g65670	*iaa9-1*	GABI_388E02
	iaa9-2	SALK_057396
At3g54830	*avt1b-1*	WiscDsLox335B10
	avt1b-2	WiscDsLoxHs144_01A
At1g10070	*bcat2-1*	SALK_037854
	bcat2-2	FLAG_353G11

Supplemental Table S5. Primers used in the present study.

Primer name	Sequence
For genotyping	
GK-767E02-F	CAATGGAAGCATTGAAAAGACGGG
GK-767E02-R	TTGTGAGATTCGTACGGACATTTG
SALK_047096/SAIL_716_G04-FP	TGGTTATCAATTTTGTTCGGG
SALK_047096/SAIL_716_G04-RP	AGAGAGAGACCTGGTAAGCGG
SALK_034026-FP	ATCTGTGCCAATTACAGGCTG
SALK_034026-RP	ATTTTGGGTGACGGGTTAAAC
SALK_121082-FP	CAGAGGTTCGTTCGCATTAAG
SALK_121082-RP	TTAATGGCGGATTTGATCATC
GABI_388E02-FP	GATACGGTTTCGAGTTTCTTCG
GABI_388E02-RP	CTCTCGCAGGAGATTGTGATC
SALK_057396-FP	TGGTGATTGGTATAAGCGCTC
SALK_057396-RP	CATCTTCGATTTCTCCATTGC
WiscDsLox335B10-FP	AAGCTAGCCCATACTCAACGG
WiscDsLox335B10-RP	TCTAACTTCAAGTGATCACACCG
WiscDsLoxHs144_01A-FP	AATGTTTCAGGCAATCGATTG
WiscDsLoxHs144_01A-RP	AAAACATTCACTCCTGCACATG
SALK_037854-FP	GAGGCTCTGCAAAAGAAGATG
SALK_037854-RP	AAATCAAGAAACATTTGGGGG
FLAG_353G11-FP	GAAGGGATGGCTGCTCTTAAC
FLAG_353G11-RP	ACTTCATCCACATGAACTGCC
CS23881-FP	AAAAAAGCAGGCTCCACCCGAAGATGAGAAACGTTATCAACC
CS23881-RP	CAAGAAAGCTGGGTCATAGCCGATGAGCAAATAATGATTG
For RT-qPCR	
TAT1-q-F	CTTGAACTCGTTGAAGAACTCTTCG
TAT1-q-R	AGAGATTCAGCTTAACCATCATTGC
THA1-q-F	GCTTGGAGAACACACATGCCAAC
THA1-q-R	AGCTTCACGCCATGTCTCTTCG
TRE1-q-F	AGGGCAATGGCTGGATTACTGG
TRE1-q-R	AGCCTTCCATGTCTCAGATTCCTC
GTE7-q-F	TCAACTCCACCTAGGAACATGGC
GTE7-q-R	TTGTTCTCTTCTCCGCACTACCC
bHLH093-q-F	AGGCTTCTTGTTCTGAGGGAGCTG
bHLH093-q-R	AGCTTCCACCATAACCTGCGTTTC
IAA9-q-F	TGTTCACCACTTTCACTCTTGGTC
IAA9-q-R	CACTAAGCATATCCTTCCCAGCAG
Avt1b-q-F	AGCCATCTCTACTCTGCTCGTTG
Avt1b-q-R	TCAGTGACATGACAAGGCCGAAG
BCAT2-q-F	TGCAACGTCTTTGTTGTCAAGGG
BCAT2-q-R	TCCGCGTAATCCCTTCAAGAATTG
